# Detection and characterization of lung cancer using cell-free DNA fragmentomes

**DOI:** 10.1038/s41467-021-24994-w

**Published:** 2021-08-20

**Authors:** Dimitrios Mathios, Jakob Sidenius Johansen, Stephen Cristiano, Jamie E. Medina, Jillian Phallen, Klaus R. Larsen, Daniel C. Bruhm, Noushin Niknafs, Leonardo Ferreira, Vilmos Adleff, Jia Yuee Chiao, Alessandro Leal, Michael Noe, James R. White, Adith S. Arun, Carolyn Hruban, Akshaya V. Annapragada, Sarah Østrup Jensen, Mai-Britt Worm Ørntoft, Anders Husted Madsen, Beatriz Carvalho, Meike de Wit, Jacob Carey, Nicholas C. Dracopoli, Tara Maddala, Kenneth C. Fang, Anne-Renee Hartman, Patrick M. Forde, Valsamo Anagnostou, Julie R. Brahmer, Remond J. A. Fijneman, Hans Jørgen Nielsen, Gerrit A. Meijer, Claus Lindbjerg Andersen, Anders Mellemgaard, Stig E. Bojesen, Robert B. Scharpf, Victor E. Velculescu

**Affiliations:** 1 The Sidney Kimmel Comprehensive Cancer Center, Johns Hopkins University School of Medicine, Baltimore, MD USA; 2 Department of Neurosurgery, Johns Hopkins University School of Medicine, Baltimore, MD USA; 3 Department of Oncology, Herlev and Gentofte Hospital, Copenhagen, Denmark; 4 Department of Biostatistics, Johns Hopkins Bloomberg School of Public Health, Baltimore, MD USA; 5 Department of Respiratory Medicine, Infiltrate Unit, Bispebjerg Hospital, Copenhagen, Denmark; 6 Department of Molecular Medicine, Aarhus University Hospital, Aarhus, Denmark; 7 Department of Surgery, Herning Regional Hospital, Herning, Denmark; 8 Department of Pathology, The Netherlands Cancer Institute, Amsterdam, The Netherlands; 9 Delfi Diagnostics, Baltimore, MD USA; 10 Department of Surgical Gastroenterology 360, Hvidovre Hospital, Hvidovre, Denmark; 11 Department of Clinical Biochemistry, Herlev and Gentofte Hospital, Copenhagen, Denmark

**Keywords:** Non-small-cell lung cancer, Small-cell lung cancer

## Abstract

Non-invasive approaches for cell-free DNA (cfDNA) assessment provide an opportunity for cancer detection and intervention. Here, we use a machine learning model for detecting tumor-derived cfDNA through genome-wide analyses of cfDNA fragmentation in a prospective study of 365 individuals at risk for lung cancer. We validate the cancer detection model using an independent cohort of 385 non-cancer individuals and 46 lung cancer patients. Combining fragmentation features, clinical risk factors, and CEA levels, followed by CT imaging, detected 94% of patients with cancer across stages and subtypes, including 91% of stage I/II and 96% of stage III/IV, at 80% specificity. Genome-wide fragmentation profiles across ~13,000 ASCL1 transcription factor binding sites distinguished individuals with small cell lung cancer from those with non-small cell lung cancer with high accuracy (AUC = 0.98). A higher fragmentation score represented an independent prognostic indicator of survival. This approach provides a facile avenue for non-invasive detection of lung cancer.

## Abstract (web summary)

DNA from tumour cells can be detected in the blood of cancer patients. Here, the authors show that cell free DNA fragmentation patterns can identify lung cancer patients and when this information is further interrogated it can be used to predict lung cancer histological subtype.

## Introduction

Lung cancer is the most lethal cancer worldwide^1^. The 5-year survival rate is <20%^2^ largely due to the late stage at diagnosis where treatments are less effective than at earlier stages, and the incidence of lung cancer continues to increase^3^. Although large randomized trials have demonstrated that lung cancer screening using chest low dose computed tomography (LDCT) decreases mortality in high-risk individuals^4,5^, LDCT remains underutilized, with <6% of at-risk individuals screened, due to concerns of potential harm from false-positive imaging results, radiation exposure, and morbidity from invasive diagnostic procedures^6–8^.

There is an urgent unmet clinical need for development of non-invasive approaches to improve cancer screening for high-risk individuals and ultimately the general population. Biomarker development for the early detection of lung cancer has broad clinical applications in screening as well as for distinguishing cancer from noncancerous pulmonary nodules on chest imaging^9^. Investigation of proteins^10–12^, autoantibodies^13^, gene expression profiles^14^ and microRNAs^15^ in the blood or airway epithelium have yielded promising biomarker candidates for early detection of lung cancer, although some may be confounded by age, inflammation from exposure to smoking, or other comorbid conditions such as autoimmune diseases, and none are approved for clinical use^15^.

**Fig. 1:**
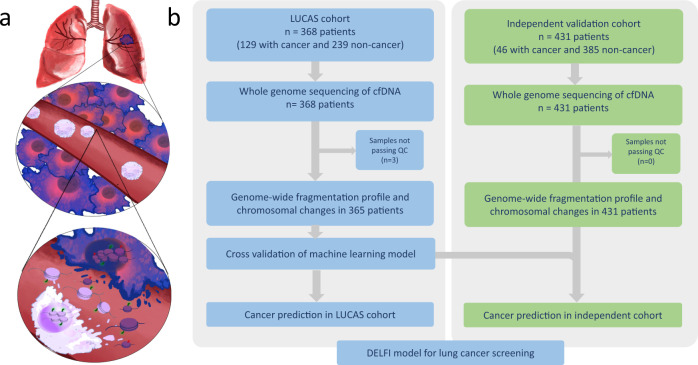
Schematic of overall approach. **a** Schematic representation of DNA fragmentation and release from apoptotic lung cancer cells and WBCs. Nucleosomal DNA with variable length of linker DNA is preserved in the circulation with cancer cell cfDNA fragments having a more aberrant profile compared to the cfDNA fragments arising from the WBCs. Mapping of the cfDNA fragments along the genome reveals distinct patterns in cancer patients compared to non-cancer individuals. **b** Outline of the DELFI approach for early detection of lung cancer. 365 patients from the LUCAS diagnostic cohort were analyzed to derive genome-wide fragmentation profiles that were used to train and evaluate the diagnostic performance in this cohort using a cross-validated machine learning model. A fixed model was used to validate the performance in an independent cohort of 46 lung cancer patients and 385 non-cancer individuals. QC, quality control.

The rapid technological and analytical advancements in liquid biopsy analyses have identified cancer-related features in the cfDNA fragments in peripheral blood and have provided a new avenue for non-invasive detection of cancer. We and others have previously shown that mutations or methylation in circulating tumor DNA (ctDNA) can be directly detected in early-stage lung cancer patients without prior knowledge of these alterations in tumors^16–21^. Given the relatively small number of sequence or epigenetic alterations that can be assessed by targeted high coverage sequencing, many individuals with cancer may be missed by such approaches and may also require sequencing of white blood cells (WBCs) to eliminate changes that result from clonal hematopoiesis^17,18,22^, although whole-genome cfDNA methylation analyses may overcome some of these issues^21^. To increase the sensitivity of detection of early-stage cancers we have developed a genome-wide approach for analysis of cfDNA fragmentation profiles called DELFI (DNA evaluation of fragments for early interception)^23^. This approach provides a view of cfDNA “fragmentomes”, permitting evaluation in any individual of the size distribution and frequency of millions of naturally occurring cfDNA fragments across the genome. As a cfDNA fragmentome can comprehensively represent both genomic and chromatin characteristics, it has the potential to identify a large number of tumor-derived changes in the circulation. In this study, we have used this methodology for lung cancer detection and characterization in a prospectively collected real-world cohort comprising patients with malignant and benign pulmonary nodules as well as non-cancer individuals, including those with other clinical conditions (Fig. 1 a, b). Through this effort, we provide a framework for incorporating non-invasive liquid biopsies in the clinic, combining cfDNA fragmentation profiles with other markers and LDCT for lung cancer detection.

## Results

We examined patient blood samples from a prospective observational trial of 365 individuals examined consecutively at Bispebjerg Hospital in Copenhagen, Denmark (LUCAS cohort) during a seven-month period. The majority of subjects in the cohort were symptomatic individuals at high risk for lung cancer (age 50–80 and smoking history >20 pack-years) (Table 1, Supplementary Table 1). The cohort included 323 subjects (90%) with pulmonary, non-pulmonary or constitutional symptoms, with the majority having common smoking-related symptoms such as cough or dyspnea. The remainder were asymptomatic at enrollment, with an incidental chest image finding by X-ray or CT that was suspicious for lung malignancy. At the time of the patient’s clinic visit, an additional chest CT or 18F-PET/CT was performed to assess the identified nodule or infiltrate (Supplementary Fig. 1). Of the 365 individuals studied, 129 were determined to have lung cancer a few days after the time of the blood collection (median 9.5 days, range 0–44) while the remainder had histologically proven benign nodules (*n* = 87) or were not biopsied due to low clinical and radiographic suspicion for cancer (*n* = 149) (Supplementary Fig. 1). Standard algorithms for the management of pulmonary nodules, including the Fleischner Society pulmonary nodule recommendations^24–26^, were used to determine clinical management.

**Table 1:** Patient demographics and clinical information in LUCAS cohort.

Patient Characteristic	Non-cancer individuals *n* = 236	Lung cancer patients *n* = 129	*P*-value*	Lung lesion histology	*n*
Age				Benign	87
Mean	63	69	<0.001	Adenocarcinoma	62
Range	19–96	33–94		Squamous cell carcinoma	29
Sex				Small cell	11
Male	125	61	0.3	Adenosquamous	3
Female	111	68		NSCLC, not otherwise specified	3
Smoking pack-years				Mixed small cell and NSCLC	1
Mean	26	42	<0.001	Mesothelioma	1
Range	0–110	0–150		Neuroendocrine	1
Never smoker	45	7	0.004	Metastasis from other organ	15
Current smoker	71	51		Unknown	3
Quit >6 months	96	55		Stage	
Quit <6 months	24	13		IA	11
Unknown	–	3		IB	4
History of cancer				IIA	2
None	183	94	0.31	IIB	5
Prior cancer (<5 yrs)	23	16		IIIA	17
Prior cancer (>5 yrs)	27	13		IIIB	15
Prior cancer (<5 yrs and >5 yrs)	4	6		IIIC	3
Prior lung cancer	2	5		IV	72

*P-values were calculated to compare data from individuals with and without lung cancer for the following variables: mean ages and smoking pack years using Student’s unpaired two-tailed *t*-tests, sex distribution using a *χ*2 test, and smoking status and history of cancer using one-way ANOVAs.

We isolated 2–4 ml of plasma from each patient in the LUCAS cohort and examined the extracted cfDNA using the DELFI approach with experimental and bioinformatic improvements. As PCR is known to affect the representation of amplified genomic fragments depending on GC content and fragment length, we evaluated DELFI genome-wide fragmentation profiles using genomic libraries created without amplification or with 4 or 12 cycles of PCR. We found that libraries created with 4 cycles of PCR had profiles that were similar to those without any amplification, while 12 cycles led to substantial biases (Supplementary Fig. 2a, b). We developed a novel fragment-based GC correction method that simultaneously accounts for preferential amplification by fragment length and/or GC content (see “Methods”). We examined whether this approach among 4 cycle libraries would further minimize GC biases compared to a commonly used bin-based approach^27^ and found that the fragment-based approach was closest to the libraries without amplification (Supplementary Fig. 2c). We therefore used a 4 cycle amplification to generate genomic libraries, and sequenced the genomic fragments using shallow whole-genome sequencing (~2x coverage) with an average of 40 million paired reads per sample (Fig. 1, Supplementary Table 2). To examine genome-wide cfDNA fragmentation patterns, we used the fragment-based GC corrected sequence data to evaluate fragmentation profiles across the genome in 473 non-overlapping 5 MB regions with high mappability, each region comprising ~80,000 fragments, and spanning approximately 2.4 GB of the genome.

**Fig. 2:**
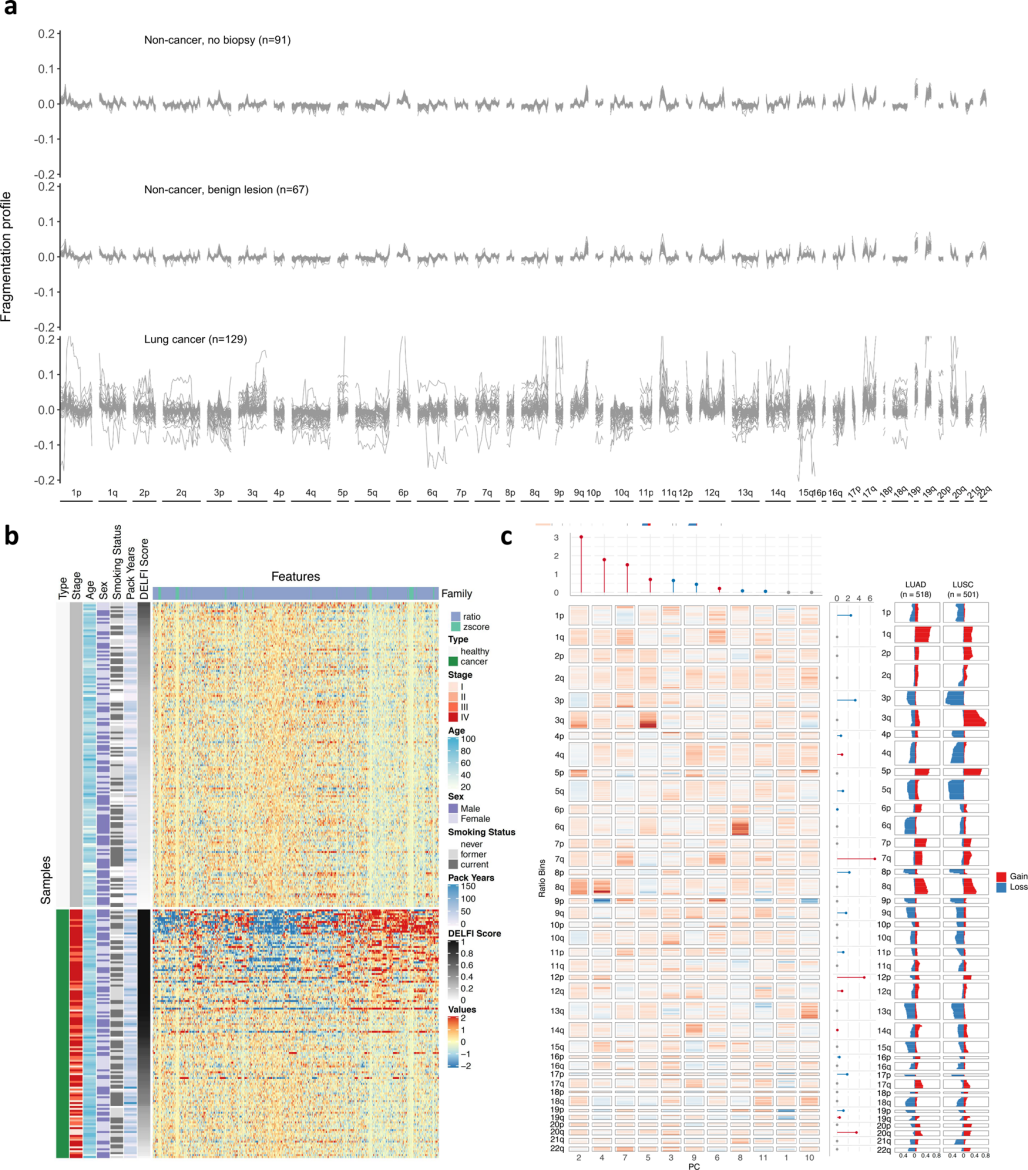
Cell-free DNA fragmentation profiles of lung cancer patients and non-cancer individuals. **a** The ratio of short to long cfDNA fragments in 5 Mb bins across the genome was evaluated in plasma samples of lung cancer and non-cancer individuals from the LUCAS cohort. The non-cancer individuals had similar fragmentation profiles while lung cancer patients exhibited significant variation. **b** Heatmap representation of the deviation of cfDNA fragmentation features across the genome for patients with lung cancer or non-cancer individuals compared to the mean of non-cancer individuals. Overall DELFI score and clinical characteristics are indicated to the left of the fragmentation deviation heatmap. **c** Heatmap representation of principal component eigenvalues of the fragmentation profile features. The relative importance of the features are shown at the top (fragmentation changes) and right (chromosomal arm changes) of the heatmap, with colors indicating increases (red) or decreases (blue) of the coefficient of cancer risk. TCGA derived observations of chromosomal arm gains (red) and losses (blue) in lung adenocarcinoma (LUAD) (*n* = 518) and squamous cell cancers (LUSC) (*n* = 501) are indicated at the right margin. Agreement between the color of the variable importance bar in LUCAS and the TCGA copy number data indicates a correspondence between higher cancer risk due to decreases (blue) or increases (red) in chromosomal arm level representation in LUCAS and copy number amplifications (red) and copy number deletions (blue) in TCGA, respectively.

The resulting fragmentation profiles were remarkably consistent among non-cancer individuals, including those with non-malignant lung nodules (Fig. 2a, b). In contrast, cancer patients displayed widespread genome-wide variation (Fig. 2a, b). Remarkably, the fragmentation profile differences could be observed in multiple regions throughout the genome for the majority of cancer patients, including across stages and histologies. We employed a machine learning model to examine whether cfDNA profiles had characteristics of an individual with or without lung cancer. Due to the high dimensionality of our genome-wide fragmentation profiles relative to the number of patients analyzed, we performed a principal component analysis (PCA) to identify linear combinations of our fragmentation features that explained at least 90% of the variance. We incorporated this dimensionality reduction step into a machine learning model and estimated the performance characteristics by repeated fivefold cross-validation, generating a score for each individual as an average over the cross-validation repeats (DELFI score). Analysis of the features incorporated in the machine learning models and corresponding measures of variable importance revealed fragmentation and chromosomal changes that were altered in cancer patients and predictive of cancer risk (Fig. 2c). The importance of these features were consistent across the training folds (Supplementary Fig. 3). Among the genomic changes incorporated in the model, chromosomal arms that were increased or decreased in cfDNA representation corresponded to those commonly gained or lost in lung cancer as seen in previous TCGA large-scale genomic studies for lung adenocarcinoma (*n* = 518) and squamous cell carcinoma (*n* = 501) (Fig. 2c). These included increased cfDNA levels of 7q, 12p, and 20q, or decreased levels of 1p, 3p, 8p, and 17p, all known to be gained or lost, respectively, in a variety of lung cancers^28–30^.

**Fig. 3:**
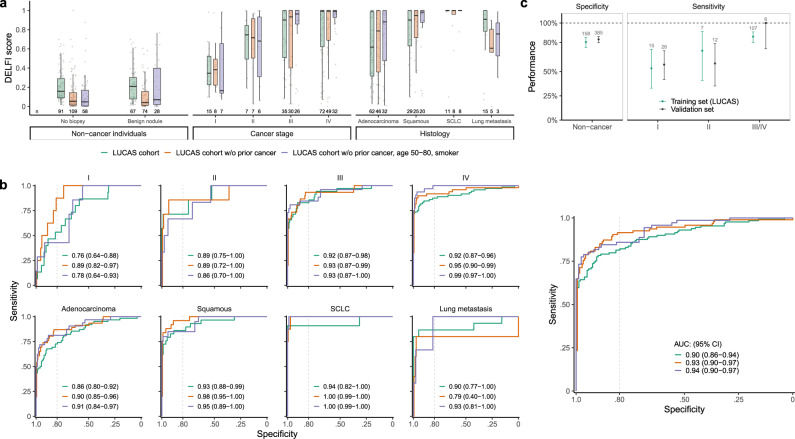
Performance of DELFI analyses for lung cancer patients and non-cancer individuals. **a** DELFI score distribution across non-cancer individuals and cancer patients, stratified by stage and histology groups in the LUCAS cohort. The box-plot shows the median DELFI score and the inter-quartile range with the individual sample values overlaid as dots. The non-cancer cases with or without benign lesions have a lower DELFI score compared to cancer cases and there is a stepwise increase in DELFI score by stage. The highest median DELFI score is observed in SCLC cases. Green curves indicate all individuals in the LUCAS cohort, orange represents patients without prior history of cancer, and blue indicates patients without prior history of cancer, age 50–80, and with ≥20 pack-year smoking history. The center line in the boxplots represents the median, the upper limit of the boxplots represents the third quantile (75th percentile), the lower limit of the boxplots represents the first quantile (25th percentile), the upper whiskers is the maximum value of the data that is within 1.5 times the interquartile range over the 75th percentile, and the lower whisker is the minimum value of the data that is within 1.5 times the interquartile range under the 25th percentile. **b** ROC analyses of the overall LUCAS cohort as well as by stage and histology. The dotted vertical line in the ROC figures represents an 80% specificity as a decision boundary. **c** Analysis of a DELFI fixed model and score cutoff of 0.344 determined from the LUCAS cohort was applied in the validation cohort. The performance of this classifier in the independent cohort was similar to LUCAS in both specificity (left) and sensitivity (right) across all tumor stages. The number of samples in the training and validation sets are indicated in the labels of the horizontal axis. The intervals presented reflect a 90% confidence interval. Additional analyses at other specificities are indicated in Supplementary Fig. 6.

As clinical characteristics may affect tumor biomarkers, we first sought to investigate whether non-malignant nodules, demographic parameters such as age or smoking history, or the presence of chronic obstructive pulmonary disease (COPD) or autoimmune diseases were associated with DELFI scores (Supplementary Table 1). An unbiased analysis of these characteristics was possible because of the prospective observational trial collection of the LUCAS cohort. We observed no difference in the DELFI score when comparing non-cancer individuals with or without benign lung lesions (median DELFI score 0.16 vs 0.21, *p* = 0.99, Wilcoxon rank sum test, Fig. 3a). We also did not observe changes in the DELFI score among age groups (*F* statistic = 1.65, *p* = 0.20), among current, prior, and never-smokers (*F* statistic = 1.3, *p* = 0.27), and across pack-years in non-cancer individuals (*F* statistic = 0.67, *p* = 0.57) (Supplementary Fig. 4). Similarly, we did not observe differences between patients with or without COPD (*p* = 0.26) or patients with or without autoimmune diseases (*p* = 0.38). Finally, we did not observe a correlation between the levels of the inflammatory markers CRP or IL-6 and DELFI score in cancer-free individuals, consistent with the notion that cancer-specific fragmentation is not affected by the presence of acute or chronic inflammatory conditions (Supplementary Fig. 5).

We next examined the relationship between DELFI scores and cancer stage and histology. While the DELFI score for non-cancer individuals was low (median DELFI scores of 0.16 or 0.21 for those without a biopsy or with benign lesions, respectively), patients with cancer had significantly higher median DELFI scores (DELFI scores for stage I = 0.35, stage II = 0.75, stage III = 0.90, and stage IV = 0.99) (*p* < 0.01 for Stages I, II, III, or IV, Wilcoxon rank sum test) (Fig. 3a). A receiver operator characteristic (ROC) curve representing sensitivity and specificity of the DELFI approach to identify cancer patients in the LUCAS cohort revealed an area under the curve (AUC) of 0.90 (95% CI = 0.86–0.94) (Fig. 3b). Stage I disease was more difficult to identify (AUC = 0.76) but stage II, III, and IV disease had similarly high performances (AUC_II _= 0.89, AUC_III _= 0.92, AUC_IV _= 0.92, respectively). When considering the detection of individuals without a prior history of cancer, consistent with the inclusion criteria of other cancer screening studies^4,5^, we observed a higher performance overall (AUC = 0.93, 95% CI = 0.90–0.97) as well as for individual stages (AUC_I _= 0.89, AUC_II _= 0.89, AUC_III _= 0.93, AUC_IV _= 0.95) (Fig. 3b). Similarly, analyses of the subset of individuals in this group that were considered at high risk for lung cancer (50–80 years old, smoking history ≥20 pack-years) revealed an overall AUC of 0.94 (Fig. 3b). Analyses of different histologic subtypes of lung cancer showed that small cell (SCLC) and squamous cell (SCC) lung cancers were more easily detected than lung adenocarcinoma (Fig. 3b). To evaluate the robustness of a prior multi-cancer DELFI approach, we examined the features and machine learning approach of Cristiano et al.^23^ in the current study and identified similar performances (AUC = 0.87, 95% CI = 0.82–0.91, for all patients, and AUC = 0.90, 95% CI = 0.86–0.94 for patients without a prior history of cancer) (Supplementary Fig. 6a). Other whole-genome analyses, such as ichorCNA^31^ which only includes copy number changes, and analyses of overall median cfDNA fragment lengths provided substantially weaker performance with overall AUCs of 0.76, (95% CI = 0.70–0.82) and 0.61 (95% CI = 0.54–0.67), respectively (Supplementary Fig. 6b).

To externally validate the predictive performance of DELFI in an independent group of individuals with or without lung cancer, we first developed a single fragmentation-based machine learning model using the non-cancer individuals and patients with baseline lung cancer in the LUCAS study and determined the DELFI score cutoff required to achieve specificities ranging from 70 to 85%. Next, we used the fixed model from LUCAS to compute DELFI scores in an independent validation cohort comprised of individuals without cancer (*n* = 385) or predominantly early-stage cancer (*n* = 46) (Supplementary Tables 3 and 4). Using the previously established cutoffs, we predicted the cancer status for individuals in the validation set according to whether their DELFI score was above or below the cutoff. The sensitivities and specificities of this model in the validation cohort were similar to those observed in the LUCAS cohort at different stages of the disease and among different histologic subtypes (Fig. 3c, Supplementary Fig. 7). Overall, these analyses suggest that the DELFI approach is generalizable across different lung cancer cohorts, including across different stages and histologic subtypes.

To evaluate multimodal approaches for cancer detection in combination with our multi-feature cfDNA analyses, we first assessed the serum levels of carcinoembryonic antigen (CEA), a secreted protein that has been proposed as a lung biomarker^12,15,32,33^ (Supplementary Table 5). Patients with lung cancer had higher CEA levels compared to patients without cancer, with more than 20% of stage I–III and the majority of stage IV cancer patients having levels >7.5 ng/ml, while only ~4% of non-cancer patients fell above this threshold^12,15,34^ (*p* < 0.001)(Supplementary Fig. 8). CEA levels increased with stage, and patients with adenocarcinoma and SCLC subtypes showed higher levels compared to those with SCC or metastases to the lung (Supplementary Fig. 8). As clinical characteristics have been proposed as risk factors for lung and other cancers^35^, we combined our genome-wide cfDNA fragmentation features with CEA levels, age, smoking history, and presence of COPD in a multimodal model (DELFI_multi_) (see “Methods”). We used repeated cross-validation to predict whether these multimodal features represented characteristics of non-cancer individuals or cancer patients. Assessment of performance of the DELFI_multi_ revealed an overall AUC of 0.93, for individual stages (AUC_I_ = 0.78, AUC_I _I= 0.95, AUC_III_ = 0.94, AUC_IV_ = 0.95), and across histologic subtypes (AUC_adeno_ = 0.91, AUC_SCC_ = 0.95; AUC_SCLC_ = 0.96) (Supplementary Fig. 9). Although we could not evaluate this approach in the validation cohort, these analyses suggest that the combination of DELFI with a serum protein and clinical risk factors may improve DELFI performance compared to those obtained through fragmentation profiles alone.

**Fig. 4:**
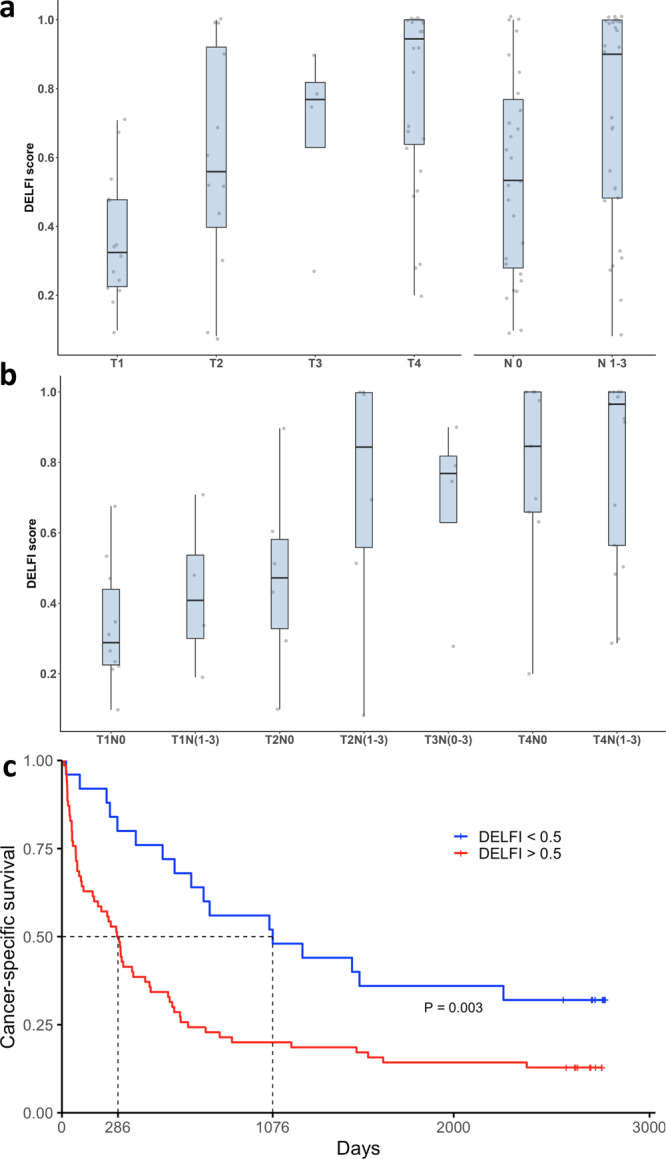
Relationship of size and invasiveness of lung cancer with DELFI score. **a** DELFI scores of non-metastatic patients with lung cancer categorized by T stage or N stage in the LUCAS cohort. We observe an incremental increase of the DELFI score by T stage from T1 to T4 (*p* < 0.01, Kruskal–Wallis, df = 3, two-sided) (*n*: T1 = 14, T2 = 12, T3 = 4, T4 = 26). Lung cancer patients without involvement of lymph nodes had a significantly lower DELFI scores compared to patients with nodal spread (Wilcoxon rank sum test, *p* < 0.001, two-sided) (*n*: N0 = 27, N 1–3 = 29). **b** The stepwise increase in DELFI score by T and N stage was maintained when considering both T and N stages in each patient (Kruskal–Wallis, df = 6, *p* < 0.01, two-sided) (*n*: T1N0 = 10, T1N(1–3) = 4, T2N0 = 6, T2N(1–3) = 6, T3N(0–3) = 4, T4N0 = 9, T4N(1–3) = 17) **c** Patients with primary lung cancer were stratified in two groups based on a DELFI cutoff of 0.5 (*n* = 93). Patients with a DELFI score > 0.5 (red) had a significantly worse cancer-specific survival compared to patients with DELFI score < 0.5 (blue) (*P* = 0.003, Log-rank test, two-sided). The center line in the boxplots represents the median, the upper limit of the boxplots represents the third quantile (75th percentile), the lower limit of the boxplots represents the first quantile (25th percentile), the upper whiskers is the maximum value of the data that is within 1.5 times the interquartile range over the 75th percentile, and the lower whisker is the minimum value of the data that is within 1.5 times the interquartile range under the 25th percentile.

To examine the relationship between fragmentation profiles and lung tumor progression we assessed whether the size of the lung cancer lesion or other clinical or radiological findings were related to aberrant fragmentation profiles. While previous studies suggest small tumors (e.g., ~1 cm^3^) may be missed by mutation-based approaches given the limited number of ctDNA molecules at specific locations and limits of detection with these methods of ~0.1%^20^, genome-wide approaches may allow for more sensitive detection of such changes. With a DELFI approach that interrogates ~40 million fragments, we would expect that ~40,000 fragments across the genome would be tumor derived in a patient with a small tumor having a 0.1% ctDNA contribution, thereby increasing the chances of detection. Interestingly, in the LUCAS cohort, eight of the nine tumors less than two cm in size (T1a) had DELFI scores higher than the median non-cancer population (median DELFI score of 0.40 vs. 0.16) (Fig. 4a). Analyses of DELFI scores and T stage in patients with localized disease showed a stepwise increase of the DELFI scores from T1 to T4 stages (median DELFI scores for T1 = 0.32, T2 = 0.56, T3 = 0.77, T4 = 0.94; T1 vs T4, *p* < 0.001, Wilcoxon rank sum test). In addition, lung cancer patients without nodal involvement (N0) had a significantly lower DELFI score compared to patients with lymph node metastases (Fig. 4a, *p* < 0.001, Wilcoxon test). A stepwise increase in DELFI scores was also observed when assessing T and N stages in affected patients (Fig. 4b, *p* = 0.005). These observations indicate a direct relationship between lung tumor size and aberrant fragmentation profiles in the circulation and suggest that even relatively small tumors may be detectable, including in cases that were undetectable using deep-targeted sequencing (~30,000×)^23^.

The long clinical follow-up of the LUCAS cohort (7–8 years) enabled an analysis of the association between DELFI scores and survival. These analyses revealed that higher DELFI scores (>0.5) were associated with a decreased overall survival compared to DELFI scores below this threshold (*P* < 0.001, Fig. 4c). In a multivariable analysis using a Cox proportional hazards model, the association of DELFI scores with survival was independent of cancer histology and stage with a hazard ratio (HR) of 2.53 (*p* = 0.001, Supplementary Fig. 11b; Supplementary Table 6). Similar results were obtained when analyzing a homogenous population of patients with stage IV adenocarcinoma (*p* = 0.004, Supplementary Fig. 11a), or when using DELFI score thresholds ranging from 0.3 to 0.9. The DELFI score remained an independent prognostic factor even when considering differences in therapy among these individuals (*P* = 0.04, HR = 2.3) or when excluding patients that had a short survival (Supplementary Table 1). These results substantiate the relationship between fragmentation patterns and tumor burden or aggressiveness, and may provide clinical insights into long-term lung cancer outcomes.

**Fig. 5:**
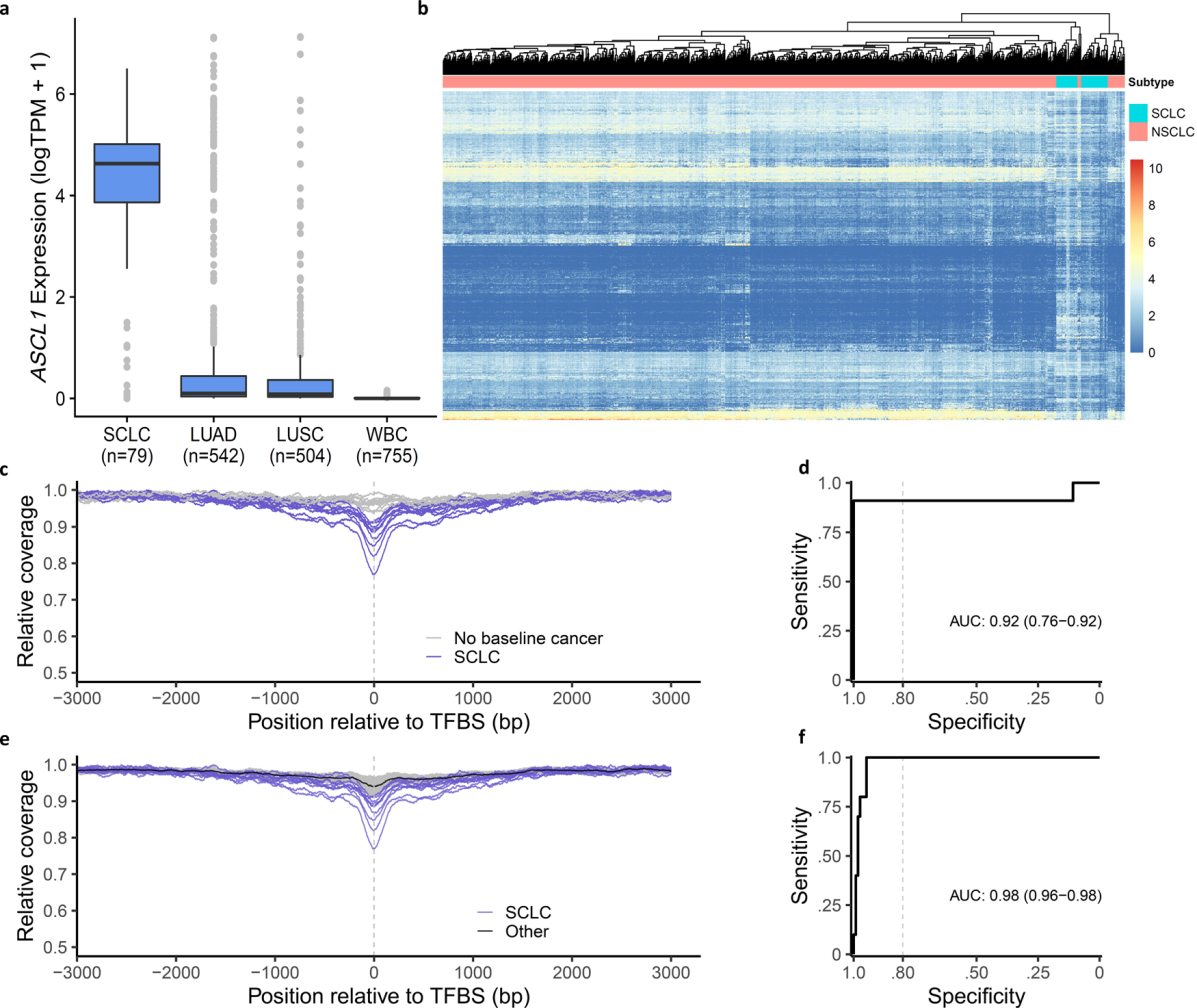
Genome-wide fragmentation profiles can distinguish SCLC from NSCLC. **a** Expression of *ASCL1* transcription factor in TCGA RNA-seq analyses of SCLCs (*n* = 79) is high compared to NSCLC (*n* = 1046) or WBC (755) samples. TPM transcripts per million. The center line in the boxplots represents the median, the upper limit of the boxplots represents the third quantile (75th percentile), the lower limit of the boxplots represents the first quantile (25th percentile), the upper whiskers is the maximum value of the data that is within 1.5 times the interquartile range over the 75th percentile, and the lower whisker is the minimum value of the data that is within 1.5 times the interquartile range under the 25th percentile. **b** Unsupervised clustering analyses of gene expression in TCGA lung cancer cohorts show that genes with ASCL1 binding sites are differentially expressed between SCLCs and NSCLCs. Genome-wide cfDNA fragmentation analyses at ASCL1 binding sites in LUCAS cohort patients reveal a decrease in coverage near transcription factor binding sites of SCLC patients compared to non-cancer individuals (**c**) or DELFI positive patients with SCLC compared to other individuals (**e**). These molecular features can distinguish SCLC patients (*n* = 11) from non-cancer individuals (*n* = 158) (**d**, AUC = 0.92) and DELFI positive SCLC patients (*n* = 10) from NSCLC patients and others (*n* = 115) (**f**, AUC = 0.98), with high accuracy.

Given the important differences in biologic characteristics and clinical management of SCLC and non-small cell lung cancer (NSCLC), we evaluated whether genome-wide fragmentation profiles could be used to non-invasively distinguish between these cancer types. We utilized publicly available TCGA RNA-seq data from lung cancer subtypes to identify transcription factors with the highest differential expression between SCLC (*n* = 79) and NSCLC (*n* = 1046) or WBC (*n* = 755) samples, and identified *ASCL1* (Achaete-Scute Family basic helix-loop-helix Transcription Factor 1) as the gene most highly differentially expressed (>960 fold compared to NSCLC and WBC) (Fig. 5a). ASCL1 is a pioneer transcription factor in neuroendocrine cells, the progenitor cell type of SCLC, and has been identified to be overexpressed in the majority of SCLCs^30^. As expected, a subset of the genes with ASCL1 binding sites were differentially expressed between SCLC and NSCLC (Fig. 5b). Given the reported differences in cfDNA coverage at regions of transcription factor binding^36^, we evaluated whether fragment coverage and size across the observed 13,693 genome-wide binding sites of ASCL1 were altered in cfDNA of SCLC patients. We observed a remarkable and consistent decrease in aggregate fragment coverage at regions containing the ASCL1 binding sites (±200 bp) of patients with SCLC compared to non-cancer individuals or those with other cancer types (Fig. 5c). In contrast, at distances further from ASCL1 binding sites (>2000 bp), the fragment coverage for patients with SCLC and other patients were similar. cfDNA fragment sizes in regions of ASCL1 binding were larger, presumably reflecting the decreased contribution of the tumor-derived cfDNA at these regions^23^ (Supplementary Fig. 10a, c). Using fragment information in the ASCL1 binding regions, we created a classifier that could be used to accurately detect 10 of 11 SCLCs (91% sensitivity, 95% CI = 65–99%) compared to 158 non-cancer individuals at >99% specificity (95% CI = 98–100%) (AUC = 0.92) (Fig. 5d). In addition, when considering DELFI positive cases (DELFI score > 0.34 corresponding to an 80% specificity), we classified SCLC patients compared to other DELFI positive cases without SCLC with high accuracy (100% sensitivity, 95% CI = 78–100% at 95% specificity, 95% CI = 90–98%, AUC = 0.98) (Fig. 5e, f). Despite the limited number of SCLC cases, these findings suggest that fragmentation profiles can reflect cell type-specific genome-wide transcription factor binding and provide a non-invasive approach for distinguishing lung cancers with different histologic subtypes.

Analyses of patients with a previous history of cancer who were in clinical remission at the time of the DELFI baseline assessment identified 25 patients, five who recurred, and four who ultimately died from this disease (Supplementary Table 7). These included three patients with head and neck cancers, one with colon cancer, and one with malignant melanoma. Patients with subsequent recurrence had significantly higher DELFI scores than those individuals without recurrence (median DELFI scores 0.65 vs 0.19, *p* = 0.005) (Supplementary Fig. 12a). In addition, patients who scored positive on the DELFI test (DELFI score > 0.34) had significantly shorter relapse-free survival (time from blood draw to relapse) compared to patients with negative DELFI test scores (*p* < 0.01, Supplementary Fig. 12b). These results support the notion that the DELFI approach can be used for the detection of disease recurrence after treatment.

In addition, the longitudinal clinical follow-up available in the LUCAS cohort enabled an analysis of fragmentation profiles in individuals who were deemed cancer-free at baseline but who developed a new cancer after baseline assessment. Of the 17 study subjects with a subsequent cancer diagnosis within two years (excluding localized skin tumors), four patients had DELFI scores >0.5 at the time of enrollment, ranging from 0.5 to 1.0 within 33–481 days after enrollment (Supplementary Tables 1 and 8). The malignancies identified comprised one case of NSCLC, as well as three non-pulmonary malignancies including chronic lymphocytic leukemia (CLL) and two B cell lymphomas. These data suggest that elevated DELFI scores may identify the emergence of cancers that were clinically undetected.

**Fig. 6:**
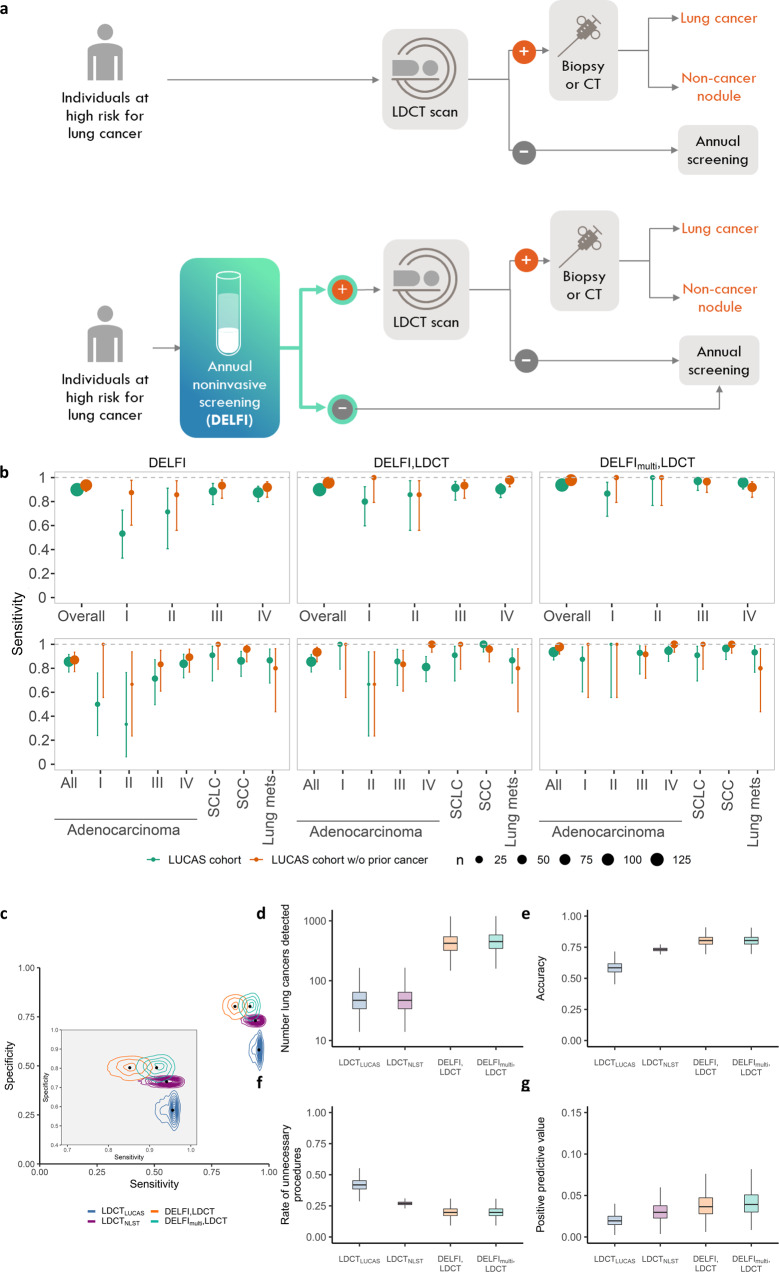
Modeling the implementation of DELFI in lung cancer screening. **a** Schematic representation of current clinical practice for lung cancer screening (top) and the proposed approach in combination with the DELFI test (bottom). In the combined approach, individuals at high-risk for lung cancer would undergo an annual blood draw that would be assessed using the DELFI test, and individuals with a positive result would subsequently undergo an LDCT scan for detection of lung cancer, while individuals with a DELFI negative result would repeat their screening annually. **b** Sensitivity of DELFI alone or DELFI followed by LDCT for lung cancer detection were compared holding specificity for the single analysis or the joint analysis at 80%. For these analyses, we considered individuals with lung cancer as those detected at baseline with LDCT, although three individuals were identified with lung cancer at a repeat LDCT within a year. The number of individuals in the LUCAS cohort are as follows: stage I *n* = 15, II *n* = 7, III *n* = 35, IV *n* = 72; and individuals in the cohort with lung adenocarcinoma comprised stage I *n* = 8, II *n* = 3, III *n* = 14, IV *n* = 37. The points colored green refer to analyses of all patients in the LUCAS cohort, whereas orange points indicate analyses of individuals without a prior history of cancer. The number of individuals are indicated schematically by the size of the dots and in Supplementary Table 1. The error bars represent the 90% confidence interval. **c** We modeled the uncertainty of sensitivity and specificity of LDCT alone as well as DELFI followed by LDCT for screening in a theoretical population of 100,000 high-risk individuals. Predictive distributions for the number of lung cancers detected (**d**), accuracy (**e**), rate of unnecessary procedures (**f**), and positive predictive values (**g**) among these individuals incorporated variation in both the prevalence of lung cancer and adherence to image- and blood-based screening. The center line in the boxplots represents the median, the upper limit of the boxplots represents the third quantile (75th percentile), the lower limit of the boxplots represents the first quantile (25th percentile), the upper whiskers is the maximum value of the data that is within 1.5 times the interquartile range over the 75th percentile, and the lower whisker is the minimum value of the data that is within 1.5 times the interquartile range under the 25th percentile.

To evaluate the theoretical impact of a non-invasive molecular blood test on lung cancer detection, we examined the performance of the DELFI score or the multimodal DELFI_multi_ score followed by standard diagnostic CT imaging in the LUCAS cohort. This would allow us to examine the scenario where high-risk individuals would first have a DELFI blood test as a prescreen and, depending on the results of the cfDNA analyses, individuals follow the pathway of either having an LDCT if the DELFI test is positive or not having an LDCT if the test is negative (Fig. 6a). Analysis of the performance of LDCT alone in the LUCAS cohort demonstrated high sensitivity (>95%) and a low specificity (58%). In a model where the DELFI score would have been used to prescreen patients, and only those that were positive were further evaluated by LDCT, the observed sensitivity of the combined DELFI/LDCT approach would be 90% (stage I = 80%, stage II = 86%, stage III = 94%, and stage IV = 90%) and provide an increase in specificity to 80% (Fig. 6b). The DELFI_multi_ approach followed by LDCT would have improved the sensitivity to 94% overall (stage I = 87%, stage II = 100%, stage III = 97%, and stage IV = 96%) at the same specificity, and would have decreased the number of unnecessary procedures from 67 with LDCT alone to 32 (52% reduction) when using the combined approach (Fig. 6b).

To examine how our approach would perform for the overall detection of individuals with lung cancer at a population scale, we evaluated the DELFI model in a theoretical population of 100,000 high-risk individuals using Monte Carlo simulations. Using the estimated sensitivities and specificities of LDCT alone or with DELFI as a prescreen in this hypothetical population (Fig. 6b), we modeled the uncertainty of these parameters using probability distributions centered at empirical estimates obtained from the NLST and/or LUCAS cohorts (Fig. 6c, Supplementary Table 9). The likely prevalence of lung cancer in this population using the NLST study^5^ estimate of 0.91% would be 910 individuals (95% CI, 428–1584). Despite the recommendations for LDCT screening, adherence in the US is only 5.9%^7^, resulting in an average of 5979 individuals tested (95% CI, 3176–9658). As blood tests offer high accessibility and compliance, with adherence rates of 80–90% reported for blood-based biomarkers^37,38^, we assumed that an average of 60% (95% CI, 39–76%) of the lung cancer screening population would be tested using the combined approach. Monte Carlo simulations from these probability distributions revealed that LDCT alone detected an average of 51 individuals (95% CI, 17–108) with lung cancer (Fig. 6d). Using DELFI as a prescreen for LDCT, we would detect on average 394 additional lung cancer cases, or an ~8-fold increase (95% CI, 4.4- to 19.6-fold increase) compared to LDCT alone (Fig. 6d). The combined approach would not only substantially improve detection of lung cancer, but would be expected to increase the accuracy of the test, reduce the number of unnecessary procedures, and increase positive predictive value (PPV) from 1.9% for LDCT_LUCAS_ and 2.6% for LDCT_NLST_ (95% CI, 0.8–3.8%) to 3.9% for DELFI and LDCT (95% CI, 1.8–7.9%, Fig. 6e–g). These analyses suggest a significant population-wide benefit for using a high-sensitivity blood-based early detection test as a prescreen to LDCT for the detection of lung cancer.

## Discussion

Overall, we describe an improved DELFI approach for genome-wide fragmentation analyses for the detection of lung cancer. We propose that facile and scalable analyses of cfDNA fragmentomes could be used to prescreen high-risk populations for lung cancer to increase the accessibility of lung cancer detection and decrease unnecessary follow-up imaging procedures and invasive biopsies. Through the analysis of the LUCAS cohort, we demonstrated that the DELFI approach can detect lung cancer across all stages and histologic subtypes compared to non-cancer individuals with or without benign lung nodules. The validation of the fixed DELFI model from the LUCAS cohort in an independent validation cohort supports the generalizability of the approach. Similar to observations with targeted sequencing approaches^16,22,39–43^, the relationship between DELFI scores and tumor progression and long-term mortality suggests that the blood-based fragmentation analyses may identify occult disease not observed by imaging, or more accurately identify the aggressiveness of the disease. The distinction between NSCLC and SCLC may allow for non-invasive characterization and treatment of lung cancer patients when tissues are not available. The identification of patients by DELFI that were only found months later to have cancer through standard diagnostic methods shows the utility of the approach for cancer detection, detection of recurrent disease, and the potential for detection of cancers at earlier stages (“stage shifting”) through lung cancer screening. The possibility of combining genome-wide multi-feature fragmentation profile analyses with a standard protein marker and clinical characteristics provides an avenue for high complexity multimodal analyses that can further increase the sensitivity of the approach.

Despite the publication of the NLST trial almost a decade ago^5^, the impact of LDCT in reducing lung cancer morbidity and mortality has been limited. Challenges for this approach have included insufficient imaging facilities and infrastructure that can screen large numbers of patients, the complexity of the medical workup that requires frequent visits and shared decision making, and repetitive radiation exposure from annual screening^44^. In addition, imaging studies detect radiographic abnormalities, not cancer, and result in biopsy-identified cancer diagnoses in only a small minority of positive scan findings, while the majority of false positive findings may drive invasive diagnostic procedures as well as ongoing patient anxiety during months or years of follow-up. Finally, while screening has been recognized as an important step for early detection of lung cancer in high-risk individuals, a significant percentage of lung cancer occurs in lower risk individuals^45^ and current USPSTF recommendations do not recommend LDCT screening for these patients due to the imbalance of harms and benefits.

Although the challenges indicated above could potentially be overcome by non-invasive fragmentation profile analyses, our study has certain limitations. For example, the majority of patients in the LUCAS cohort presented with symptoms that are not fully representative of a screening population. Although analyses of the predominantly early-stage validation cohort and the high-risk smoking population in the LUCAS cohort resulted in high performance, a large prospective validation in a screening population will be necessary before clinical use. A few patients with late-stage disease were not detected by our cfDNA analyses, presumably due to ctDNA variation in lung cancer patients from the effects of histology, tumor size, and other characteristics, but the inclusion of other biomarkers as demonstrated in the multimodal DELFI approach could mitigate this limitation. In addition, the effect of the DELFI score on survival observed in our analyses may not be representative in patients treated with more recent lung cancer therapies, including immune checkpoint blockade. Nevertheless, the analyzed cohorts represent real-world, prospective populations, and the collection and processing of all samples were performed in a systematic fashion, ensuring homogeneity of pre-analytical characteristics and careful control of experimental and analytical variables. The potential improvement of the PPV in the combined LDCT/DELFI approach suggests that many fewer unnecessary procedures would be performed in individuals with positive results. In addition, the DELFI score appears to not be affected by non-cancer conditions, which have confounded other potential biomarkers for lung cancer detection. The observations that scalable and cost-effective non-invasive cfDNA fragmentation analyses can discriminate lung cancer patients from non-cancer individuals may ultimately provide an opportunity to evaluate not only high-risk individuals but the general population for lung cancer.

## Methods

### Study population analyzed

The LUCAS cohort represents a prospectively collected group of 368 predominantly symptomatic patients that presented in the Department of Respiratory Medicine, Infiltrate Unite, Bispebjerg Hospital, Copenhagen with a positive imaging finding on a chest X-ray or a chest CT. Patients diagnosed with cancer with known active disease or who were under treatment at the time of enrollment were excluded. The study was conducted over 7 months from September 2012 to March 2013, and all patients had a clinical follow-up until death or April 2020. All patients provided written informed consent and the studies were performed according to the Declaration of Helsinki. The LUCAS study was approved by the Danish Regional Ethics Committee and the Danish Data Protection Agency. All patients had blood samples collected at their first clinic visit before the possible diagnosis of lung cancer was made. Samples from 365 patients that passed quality control from genomic sequencing were included in subsequent analyses. The analyzed cohort included 158 patients with no prior, baseline, or future cancers, 129 patients with baseline lung cancer, and 78 patients without cancer at the time of blood collection, but with either earlier or later cancers (Supplementary Fig. 1, Table 1). The validation cohort consisted of samples from different sources. These included 385 non-cancer individuals from two screening clinical trial cohorts for colorectal cancer in Denmark (Endoscopy III) and the Netherlands (COCOS, Netherlands Trial Register ID NTR1829^46^). The protocol for the Endoscopy III Project was approved by the Regional Ethics Committee and the Danish Data Protection Agency, and for the COCOS trial, ethical approval was obtained from the Dutch Health Council. The inclusion criteria for both the Dutch and the Danish cohorts were any individuals of age 50–75 eligible for colorectal cancer screening. All patients used had either a FIT negative test or a negative colonoscopy result. In addition, we included 46 patients with pathologically confirmed predominantly early-stage lung cancer from an independent prospective collection through BioIVT (Westbury, NY) (Supplementary Table 4). The plasma samples of the 46 lung cancer patients included in the validation cohort originated from patients at risk for lung cancer at the time of blood collection that were identified to have a newly diagnosed lung cancer upon further diagnostic workup.

### Sample collection and preservation

The sample collection for the LUCAS cohort was obtained at the time of the screening visit and performed as follows: venous peripheral blood was collected in one K2-EDTA tube and two serum gel tubes. Within two hours from blood collection tubes were centrifuged at 2330 × *g* at 4 °C for 10 min. After centrifugation, EDTA plasma and serum were aliquoted and stored at −80 °C for cfDNA and protein analyses, respectively.

For the validation cohort, venous peripheral blood for each individual was collected in one EDTA tube. Tubes from the COCOS and the Endoscopy III as well as the BioIVT collections were centrifuged at low speed (1500–3000 g) for 10–15 min within two hours from blood collection. The plasma portion from the first spin was spun a second time for 10 min. After centrifugation EDTA plasma was aliquoted and stored at −80 °C for cfDNA analyses.

### Sequencing library preparation

Circulating cell-free DNA was isolated from 2 to 4 ml of plasma using the Qiagen QIAamp Circulating Nucleic Acids Kit (Qiagen GmbH), eluted in 52 μl of RNase-free water containing 0.04% sodium azide (Qiagen GmbH), and stored in LoBind tubes (Eppendorf AG) at −20 °C. The concentration and quality of cfDNA were assessed using the Bioanalyzer 2100 (Agilent Technologies).

Next-generation sequencing (NGS) cfDNA libraries were prepared for WGS using 15 ng cfDNA when available, or the entire purified amount when <15 ng. For the validation cohort, available cfDNA up to 125 ng was used as input material for library preparation. In brief, genomic libraries were prepared using the NEBNext DNA Library Prep Kit for Illumina (New England Biolabs (NEB)) with four main modifications to the manufacturer’s guidelines: (i) the library purification steps use the on-bead AMPure XP (Beckman Coulter) approach to minimize sample loss during elution and tube transfer steps; (ii) NEBNext End Repair, A-tailing and adaptor ligation enzyme and buffer volumes were adjusted as appropriate to accommodate on-bead AMPure XP purification; (iii) Illumina dual index adaptors were used in the ligation reaction; and (iv) cfDNA libraries were amplified with Phusion Hot Start Polymerase. All samples underwent a 4 cycle PCR amplification after the DNA ligation step.

In total, 23 batches of cfDNA library preparations were performed for the LUCAS cohort. Each batch included a combination of cancer patients and non-cancer controls (Supplementary Tables 10, 11). All batches included a technical replicate of nucleosomal DNA obtained from nuclease-digested human peripheral blood monocytes (PBMCs) to assess sequencing consistency and reproducibility across batches performed on a different date. We periodically included a negative library control where buffer TE pH 8.0 was used instead of a DNA sample to ensure there was no DNA contamination during the library preparation. The validation cohort was prepared as above and the 485 samples, including cases and controls, were spread over 33 batches (Supplementary Table 4). For the external validation of the approach, the 33 batches in the validation cohort were processed at temporally distinct times from the batches in the LUCAS cohort and by different laboratory technicians.

### Low coverage whole-genome sequencing and alignment

Whole-genome libraries of cancer patients and cancer-free individuals were sequenced using 100-bp paired-end runs (200 cycles) on the Illumina HiSeq2500 platform at 1–2x coverage per genome. Prior to alignment, adapter sequences were filtered from reads using the fastp software^47^. Sequence reads were aligned against the hg19 human reference genome using Bowtie2^48^ and duplicate reads were removed using Sambamba^49^. Post-alignment, each aligned pair was converted to a genomic interval representing the sequenced DNA fragment using bedtools^50^. Only reads with a mapq score of at least 30 or greater were retained. Read pairs were further filtered if overlapping a problematic region provided by the Duke Excluded Regions blacklist (https://genome.ucsc.edu/cgi-bin/hgTrackUi?db=hg19&g=wgEncodeMapability). To capture large-scale epigenetic differences in fragmentation across the genome estimable from low-coverage whole-genome sequencing, we tiled the hg19 reference genome into non-overlapping 5 Mb bins (Supplementary Table 12). Bins with an average GC content <0.3 and an average mappability <0.9 were excluded, leaving 473 bins spanning approximately 2.4 GB of the genome (Supplementary Table 11).

### Whole-genome fragment features

Ratios of the number of short (100–150 bp) to long (151–220 bp) fragments across the 473 bins were normalized for GC-content and library size. As GC-related biases in coverage have been largely attributed to preferential amplification of fragments during PCR^27^, we developed a non-parametric method for fragment-level GC adjustment. For each individual in the LUCAS cohort, we assigned each fragment to one of 100 possible GC strata between 0 and 1 (1 indicating a fragment with all G and C nucleotides), obtaining the total number of fragments within each GC stratum. In the same manner, we obtained a distribution of fragment counts by GC stratum for the held-out set of 54 non-cancer samples (Supplementary Table 2) as well as the median of the 54 distributions that we refer to as a target distribution. To normalize sample-to-sample PCR biases in LUCAS, the collection of fragments in GC stratum *i* for a sample in the LUCAS cohort were assigned a weight *w*_*i*_ such that$\mathop{\sum }\limits_{i=1}^{{N}_{i}}{w}_{i}={t}_{i}$, where *t*_*i*_ denotes the number of fragments in the target distribution, *N*_*i*_ the total number of fragments in stratum *i*, and *i* =1, …100. We computed the GC-adjusted number of short and long fragments for each 5 Mb bin as the sum of the weights for the fragments aligned to that bin, thereby normalizing both sample-to-sample variation in GC-biases as well as differences in library size. Fragmentation profiles of the GC-adjusted short to long ratios were standardized to have mean zero and unit standard deviation across the genome.

In addition to fragmentation profiles, we also computed z-scores for chromosomal arms and a genome-wide summary of the overall cfDNA fragmentation as previously described^23,51^ with the modifications indicated below. We only analyzed the 39 chromosomal arms that were not acrocentric. Z-scores for each of the 39 autosomal arms were obtained by centering and scaling the total GC-adjusted fragment count for each arm by the mean and standard deviation of the corresponding arm-specific counts in the 54 non-cancer samples used as a reference set. In addition, using the published methodology^31^ we calculated the ichorCNA score for each sample (Supplementary Table 2).

### Analysis based on public databases from TCGA

Copy number data from the two lung cancer cohorts in TCGA (LUAD *n* = 518 and LUSC *n* = 501) were retrieved using the package RTCGA v1.16.0^52^. The tumor to normal log copy ratio values were compared to tumor type-specific thresholds^53^ to identify genomic regions harboring copy gains and losses. The copy number status in each of the 5 Mb bins across the genome was determined by requiring a minimum coverage of 90% of the bin interval by segments harboring a gain or loss. The frequency of copy gain and loss in the genomic bins were calculated for each of the lung cancer cohorts in TCGA.

### Machine learning and cross-validation analyses

We used fivefold cross-validation to develop a predictive model for early and late-stage cancer detection where feature selection and model development were evaluated on four of the five folds (training set) and a fifth held-out fold was used only to assess model performance (test set). The total number of samples available for training and testing includes 158 participants with no prior, baseline or future cancer and 129 patients with cancer at the time of the blood draw. Due to the high dimensionality of the fragmentation features relative to the number of available samples for training, we performed a PCA within each training set to reduce the dimensionality of the feature space, retaining the minimum number of principal components needed to explain 90% of the variance of the fragmentation profiles between samples. In addition to the principal component features, we evaluated all 39 z-scores in a logistic regression model with a LASSO penalty. The optimized LASSO penalty of 0.0017 in our analysis was obtained by resampling using the caret R package. The DELFI score derived for each sample corresponds to the mean score across the 10 cross validation repeats. To characterize the stochasticity of our classifier across the 50 training sets (five-folds × 10 repeats) used to derive the mean score, we saved the regression coefficients from each model (Supplementary Fig. 3).

To externally validate model performance, we first obtained a final model using all 158 non-cancer individuals and 129 patients with baseline lung cancer in the LUCAS study and determined the cutoffs that achieved specificities of 70–85%. Next, in an independent cohort (validation set) of non-cancer individuals (*n* = 385) and predominantly early-stage cancer (*n* = 46) we computed the fragmentation features needed to compute a risk score. For analyses where the DELFI result was indicated as positive or negative, the DELFI score cut-off of 0.34 with an 80% specificity was used as a decision boundary. The z-scores in the validation set were computed as previously described. For the fragmentation features, we projected the matrix of mean-centered, scaled, and GC-corrected short fragments to long fragments (*X*) onto the principal components used in the final model from the LUCAS cohort. Denoting the 473 × 11 matrix of loadings (eigenvalues) for PCs 1 through 11 in the final LUCAS model by *L*, the feature for principal component k in sample j of the validation set is given by the following formula.

Let *X* denote the matrix of mean-centered, scaled, and GC-corrected short fragments to long fragments for sample in the validation cohort with rows corresponding to genomic bin and columns corresponding to sample (dimension 473 × 43) and L the 473 × 11 matrix of loadings (eigenvalues) from the PCA used to obtain the final model in the LUCAS cohort. The feature for principal component k in sample j of the validation set is obtained by projecting *X* onto *L*:

1 $${{{{{\mathrm{P{C}}}}}}}_{k}=\mathop{\sum }\limits_{i=1}^{473}{L}_{i,k}{x}_{i,j}.$$

Multiplying the features by the regression coefficients from the fixed model, we obtained a prediction score, or log odds of cancer, for each individual in the validation set and classified these individuals as non-cancer or cancer according to whether the log odds >*θ*. As previously described the samples from the validation cohort were entirely batch-independent from the LUCAS cohort with respect to sample collection, library preparation, and sequencing (Supplementary Tables 4, 10).

To evaluate the sensitivity of our approach to individuals with a prior history of cancer or future cancer, we repeated the previously described cross-validation procedure in a cohort where prior cancer were excluded from the analysis and patients with future cancer were considered non-cancer individuals given they had no clinically detectable cancer at baseline. This scenario recapitulates the sample allocation in a prospective assessment of a screening cohort. Finally, we trained a classifier limited to individuals age 50–80 with a history of smoking ≥20 pack-years.

To assess whether clinical and serum protein markers in addition to fragmentation features could further improve prediction, we evaluated a multimodal predictive model using the repeated fivefold cross-validation approach. Fragmentation features summarized by a PCA and z-scores were evaluated as described above such that both feature selection and estimation of model parameters were independent of the test set. For clinical and serum protein markers, we included age, smoking history, COPD status, and CEA. A logistic regression model with a LASSO penalty was used to evaluate the fragmentation, clinical, and protein biomarker features in each training fold.

Credible intervals for sensitivity estimates were based on a beta-binomial model using a non-informative beta prior distribution with shape parameters of 0.5.

### Treatment in the LUCAS cohort

All patients were evaluated after diagnosis for eligibility for either (1) primary surgery, (2) concomitant chemotherapy and radiotherapy with curative intent, (3) standard palliative systemic oncological treatment (with either chemotherapy or targeted therapy), or (4) best supportive care—all according to the Danish national treatment guidelines for lung cancer in 2012–13, which were in concordance with the ESMO guidelines^54–56^.

All patients were evaluated for possible primary surgery based on TNM-stage as well as possible co-morbidities that might prevent the possibility for anesthesia. Patients underwent primary lung surgery for a solitary lung metastasis (two colorectal, one testis cancer, and one breast cancer), and a subset received post-surgery adjuvant chemotherapy according to ESMO guidelines in 2012–2013. If patients were not eligible for primary surgery, they were then evaluated at a multi-disciplinary team conference for concomitant chemotherapy (platinum doublet combined with either Vinorelbine (for NSCLC) or Etoposide (for SCLC)) and radiotherapy (either 2 Gray in 33 fractions, 5 F/W or stereotactic radiotherapy 15 Gray in 3 fractions (for NSCLC) or 2.05 Gray in 45 fractions or 3 Gray in 10 fractions (for SCLC)) with curative intent. Patients with poor ECOG performance status and/or significant co-morbidities were precluded from having any type of oncological treatment and were referred for supportive care. Patients with advanced disease at the time of diagnosis were eligible for palliative chemotherapy and/or radiotherapy. Patients with an EGFR mutation were primarily treated with Gefitinib in first line and Erlotinib following either Gefitinib or chemotherapy and those with ALK-translocation were treated with crizotinib. Patients from the initial palliative treatment cohort went on to receive second line oncological treatment after the progression of disease (typically Pemetrexed monotherapy). Only 16 patients received additional therapy after a second line treatment, with one patient receiving a total of seven lines of treatment. Since the cohort is from 2012 to 2013 only two patients received immunotherapy (Nivolumab) in respectively 3rd and 7th line in a CheckMate protocol.

### Association of clinical covariates and survival with the DELFI score

We performed univariate analyses comparing the distribution of the DELFI score to baseline clinical and laboratory covariates age, smoking, and serum inflammatory markers using a Wilcoxon rank sum test. In addition, we evaluated the relationship of the DELFI score and cancer risk with and without baseline covariates age, smoking, and sex using logistic regression.

To assess whether the DELFI score was associated with prognosis, we categorized high-risk lung cancer patients according to whether they were more likely to have cancer than not (DELFI score > 0.5). To assess whether this categorization was associated with survival among lung cancer patients in a univariate analysis, we used a log rank test to compare survival curves (Supplementary Table 6). While we did not optimize the 0.5 cutoff, we verified that inference from the log rank test was robust to other possible choices that would achieve higher or lower specificities, including a range of DELFI score cutoffs of 0.3–0.95. In addition to the univariate analysis, we evaluated whether the DELFI score was independently associated with cancer-specific survival in a multivariable Cox proportional hazards model that included age, histological subtypes of primary lung cancer, clinical staging, as well as treatment modality.

### Genome-wide transcription factor analyses for prediction of histological subtype

Gene expression values were obtained as raw counts using recount3 1.0.2^57^ and converted to transcripts per million (TPM) using recount 1.16.1 for SCLC (*n* = 79)^58^, lung adenocarcinoma (*n* = 542), and lung squamous cell carcinoma (*n* = 504) generated by The Cancer Genome Atlas (TCGA), and whole blood (*n* = 755) generated by the Genotype-Tissue Expression (GTEx) project. The median TPM value was computed for 1639 transcription factors (TFs)^59^ in each cancer/tissue type. We identified TFs that were unexpressed (median TPM < 1) in lung adenocarcinoma, lung squamous cell carcinoma, and whole blood, and then ordered them from highest to lowest expression in SCLC. The top gene was *ASCL1* (median TPM = 101). We then obtained chromatin immunoprecipitation followed by sequencing (ChIP-seq) peaks for *ASCL1* (*n* = 13,920 peaks) (GEO Sample accession number: GSM3704421)^60^. For each peak in the autosomes (*n* = 13,693 peaks) we defined the center of the peak as position 0 and then computed the coverage in a ±3000 bp window around each peak separately for 125 samples with a DELFI score of at least 0.37 (corresponding to a specificity of 85%). We excluded a small number of peaks with an average coverage of >3 across samples. The mean of the coverages at each position (−3000 to +3000) across all peaks was computed for each sample. For Fig. 5c, e the relative coverage for a given sample is computed by taking the coverage at each position in the ±3000 bp window surrounding the ASCL1 binding sites and dividing by the maximum within that sample. The SCLC samples are plotted separately, and the samples in the ‘Other’ group are plotted as the median relative coverage or fragment length (black line) and the 0.05 and 0.95 quantiles of the relative coverage or fragment length at each position relative to the ASCL1 binding sites (shaded region). For the ROC curves (Fig. 5d, f), relative coverage was computed for each sample as the mean coverage in a ±100 bp window surrounding the center of the ASCL1 binding sites divided by the mean coverage in a ±250 bp window surrounding 2750 bp upstream and downstream of the binding sites. The ROC curve was generated using pROC 1.16.2^61^. For Fig. 5b, names of 620 ASCL1 target genes was obtained^62^, 600 of which had a matching gene name in our gene expression datasets. For these 600 genes, we defined a gene as ‘Overexpressed’ in a given sample when TPM value >3 standard deviations above the mean for that gene across all samples.

Using fragments <200 bp, the mean fragment size was computed at each position in a ±3000 bp window surrounding the ASCL1 binding sites. For Supplementary Fig. 10a, the SCLC samples and 10 random no baseline cancer samples are plotted separately and smoothed using the LOESS method. For Supplementary Fig. 10a, c the relative fragment size for a given sample is computed by taking the average fragment size at each position in the ±3000 bp window surrounding the ASCL1 binding sites and dividing it by the minimum within that sample. For Supplementary Fig. 10c, the SCLC samples are plotted separately and smoothed using the LOESS method, and the samples in the ‘Other’ group are plotted as the median relative fragment size (black line) and the 0.05 and 0.95 quantiles of the relative fragment size at each position relative to the ASCL1 binding sites (shaded region). For ROC curves (Supplementary Fig. 10b, d), relative fragment length was computed for each sample as the mean fragment size in a ±100 bp window surrounding the center of the ASCL1 binding sites divided by the mean fragment size in a ±250 bp window surrounding 1250 bp upstream and downstream of the binding sites.

### Modeling of DELFI performance in a screening population

To assess the performance of LDCT alone and DELFI followed by LDCT in a hypothetical screening population of 100,000 individuals, we used Monte Carlo simulations to capture the uncertainty of unknown parameters sensitivity, specificity, adherence, and lung cancer prevalence. Prior models of sensitivity for LUCAS alone were centered loosely on empirical estimates from the LUCAS and NLST cohorts:

2 $$\begin{array}{ll}{\theta }_{1,M} & \sim \left\{\begin{array}{l}\pi \times N(0.96,0.005)+(1-\pi )\times N(0.94,0.02)M\\ {{{{\rm{Beta}}}}}(93.8,6.2)M\\ {{{{\rm{Beta}}}}}(85,15)M\,\\ {{{{\rm{Beta}}}}}(91,9)M\end{array}\begin{array}{ll}= & {{{{{{\rm{LDCT}}}}}}}_{{{{{{\rm{LUCAS}}}}}}}\\ = & {{{{{{\rm{LDCT}}}}}}}_{{{{{{\rm{NLST}}}}}}}\\ = & {{{{\rm{DELFI}}}}},{{{{\rm{LDCT}}}}}\\ = & {{{{{{\rm{DELFI}}}}}}}_{{{{{{\rm{multi}}}}}}},{{{{\rm{LDCT}}}}}\end{array}\right.\end{array}$$

We sampled $$\pi \sim {{{{{\rm{Bernoulli}}}}}}(0.5)$$.

For specificity, prior models were

3 $${\theta }_{2,M}\sim \left\{\begin{array}{rcl}{{{{\rm{Beta}}}}}(58,\,42)\,M & = & {{{{{{\rm{LDCT}}}}}}}_{{{{{{\rm{LUCAS}}}}}}}\\ {{{{\rm{Beta}}}}}(93.8,\,6.2)M & = & {{{{{{\rm{LDCT}}}}}}}_{{{{{{\rm{NLST}}}}}}}\\ {{{{\rm{Beta}}}}}(86,14)M & = & {{{{\rm{DELFI}}}}},{{{{\rm{LDCT}}}}}\\ {{{{\rm{Beta}}}}}(94,6)M & = & {{{{{{\rm{DELFI}}}}}}}_{{{{{{\rm{multi}}}}}}},{{{{\rm{LDCT}}}}}\end{array}\right.$$

The number of individuals screened in our simulated screening study depends on adherence to screening guidelines. Letting *n* denote the size of our screening study, our sampling model for *n* is given by

4 $$n \sim {{{{{\rm{Binomial}}}}}}({10}^{5},\eta )$$

$$\eta \sim {{{{{\rm{beta}}}}}}({a}_{n,}{\beta }_{n})$$

For LDCT alone, shape parameters *a*_*n*_ and *β*_*n*_ were 12 and 188^7^ while for DELFI_multi_ followed by LDCT shape parameters were 15 and 11^37^. Conditional on the size of our screening study and draws of and ${\theta }_{2,M}$ from their respective prior distributions, we sampled the disease status, *y*, and screening results, *x*, conditional on *y*:

5 $$\begin{array}{lll}{y}_{i} & \sim & {{{{\rm{Bernoulli}}}}}(\psi )\,for\,i=1,\ldots ,n\\ \psi & \sim & {{{{\rm{Beta}}}}}(9.1,990.9)\\ {x}_{i}|\{{y}_{i}=1,\,M\} & \sim & {{{{{\rm{Bernoulli}}}}}}({\theta }_{1,M})\\ {x}_{i}|\{{y}_{i}=0,\,M\} & \sim & {{{{{\rm{Bernoulli}}}}}}(1-{\theta }_{2,M})\end{array}$$

The informative prior for prevalence, $\psi$, in our hypothetical population ensures that our screening study will be comprised predominantly of individuals without cancer, but allows the true prevalence to be smaller or larger than the estimate of 0.91% from the NLST study^5^. The number of patients with lung cancers detected, accuracy, false-positive rate, and PPV were calculated from the joint distribution of *x* and *y*. We repeated the above sampling procedure 10,000 times, thereby obtaining predictive distributions for these statistics that reflect the uncertainty of sensitivity, specificity, adherence, and prevalence.

### Bioinformatic and statistical software

All statistical analyses were performed using R version 3.6.1. After trimming of adapter sequences using fastp (0.20.0), we used Bowtie2 (2.3.0) to align paired end reads to the hg19 reference genome. PCR duplicates were removed using Sambamba (0.6.8) and the remaining aligned read pairs were converted to a bed format using Bedtools (2.29.0). We used the R package data.table (1.12.8) for manipulation of tabular data and binning fragments in 5 Mb windows along the genome. The R packages caret (6.0.84) and gbm (2.1.5) were used to implement the classification by gradient boosted trees and resampling.

### Statistics and reproducibility

Computer code, software versions, and the computing environment for reproducing results from this study are provided as a GitHub repository (https://github.com/cancer-genomics/reproduce_lucas_wflow).

### Reporting summary

Further information on research design is available in the Nature Research Reporting Summary linked to this article.

## Supplementary information

Supplementary Information

Peer Review File

Description of Additional Supplementary Files

Supplementary Data 1-12

Reporting Summary

## Data Availability

Sequence data and clinical variables generated in this study have been deposited at the database of European Genome-Phenome Archive (EGA) under accession code: EGAS00001005340. The publicly available gene expression measurements from RNA-seq data used in this study can be accessed using the create_rse function in version 1.0.2 of the recount3 R/Bioconductor package [https://bioconductor.org/packages/release/bioc/html/recount3.html] by providing the project IDs SRP045225 (for SCLC), LUAD (for lung adenocarcinoma samples generated by TCGA), LUSC (for squamous cell lung cancer samples generated by TCGA), and BLOOD (for whole blood samples generated by GTEx). The raw sequencing data for the SCLC samples used in this study is available through the Sequence Read Archive using accession number SRP045225. Access to primary sequencing data generated by GTEx and TCGA can be obtained through dbGaP using accession numbers phs000424 and phs000178. The publicly available ChIP-seq data used in this study are available in the GEO DataSets database under accession code GSM3704421. Segmented copy number data, determined by analysis of the Affymetrix genome-wide human SNP array 6.0, were retrieved from Broad Institute TCGA Genome Data Analysis Center, (2016-01-28 release date, using RTCGA package, version 1.16.0). The remaining data are available within the Article, Supplementary Information or Source Data file.

## Code availability

Scripts for reproducing tables and figures in the manuscript are available in the following GitHub repository (https://github.com/cancer-genomics/reproduce_lucas_wflow) under the GNU GENERAL PUBLIC LICENSE Version 3.
