# Supplementary material for: Detection and characterization of lung cancer using cell-free DNA fragmentomes
Source: Nat Commun. 2021 Aug 20;12:5060. doi: 10.1038/s41467-021-24994-w (PMC8379179; doi:10.1038/s41467-021-24994-w)
Supplement: Supplementary file 1 — Supplementary Information [file 41467_2021_24994_MOESM1_ESM.pdf]

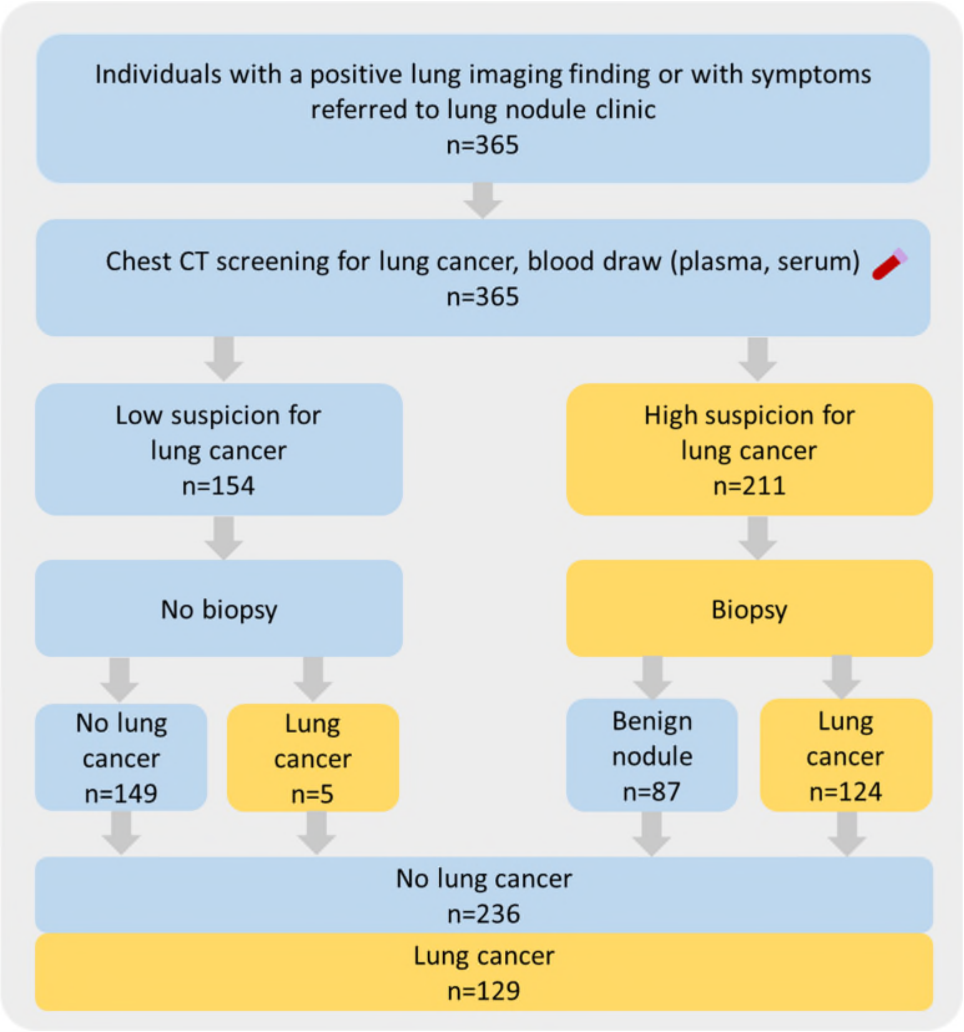

Supplementary Fig. 1. Diagnostic algorithm for the samples analyzed from the LUCAS diagnostic cohort. Patients in the LUCAS cohort were referred for a diagnostic workup after a positive finding on a chest X-ray or chest CT. All patients received a plasma and serum blood draw at the time of clinic visit as well as a chest CT to confirm the original imaging finding. The patients were stratified into low suspicion and high suspicion for lung cancer groups. Patients with low suspicion (n=150) were followed clinically and radiographically for up to 7 years. 145 of these patients had not developed lung cancer at the time of last follow-up whereas 5 of these patients had lung cancer diagnosed based on clinical history and imaging characteristics and were confirmed to have lung cancer on autopsy. Patients with high suspicion (n=208) underwent additional workup including PET-CT scan and a lung biopsy. 87 of these patients were diagnosed with a non-malignant nodule whereas 124 patients had a histologically confirmed lung cancer by biopsy.

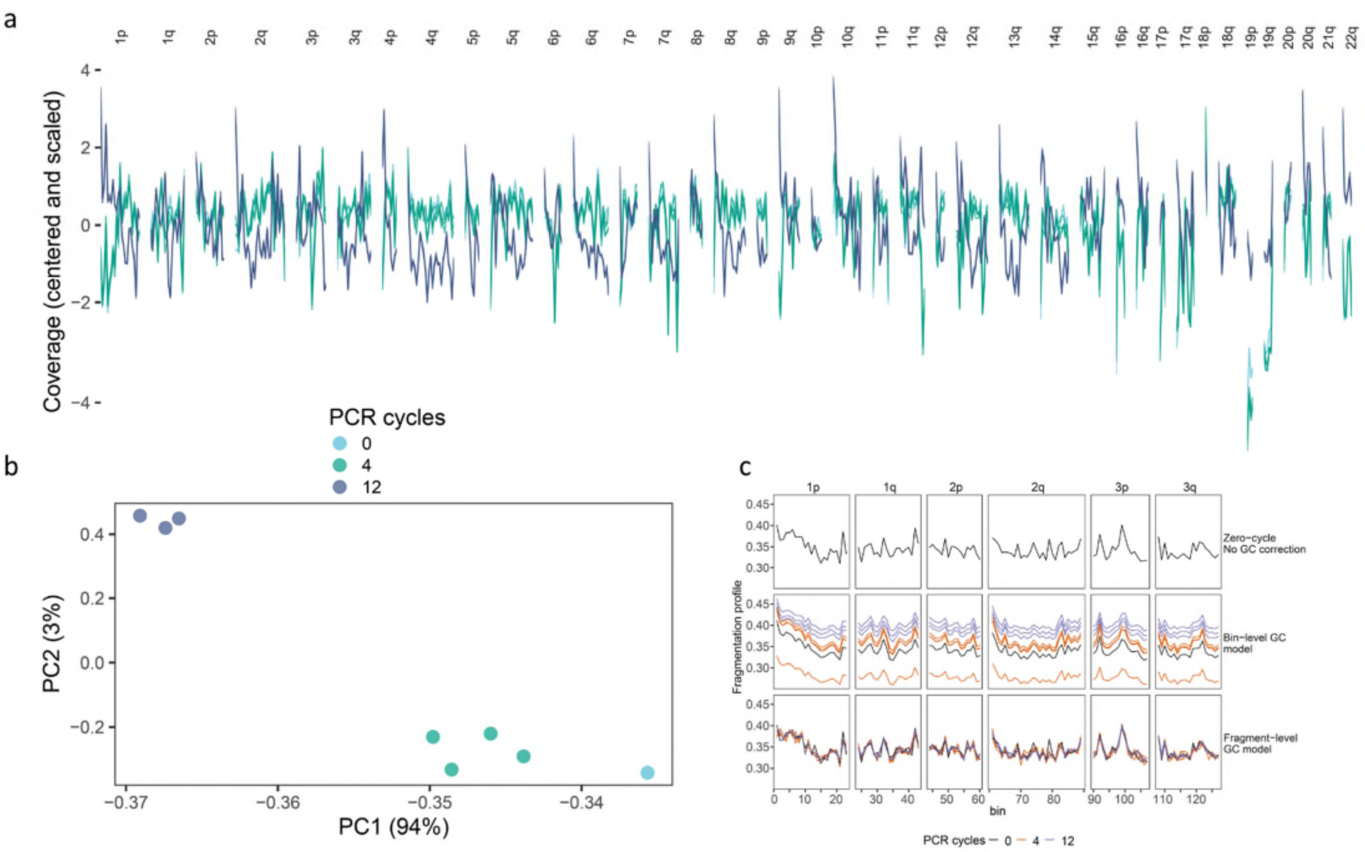

Supplementary Fig. 2. Fragmentation profiles in matched DNase digested lymphocyte DNA with various cycles of PCR amplification. a, Standardized fragment coverage of the same sample without amplification (0 cycles PCR, n=1, blue) compared to technical replicates of 4 cycles of PCR (n=4, green) and 12 cycles of PCR (n=3, grey). There were minimal effects of 4 cycles of PCR compared to no amplification, while 12 cycles were visibly different. **b**, Principal component (PC) analyses of fragmentation profiles without GC correction highlights the similarity of 0 and 4 cycle PCR fragmentation profiles. **c**, Fragment-level GC correction of sequences from 4 or 12 cycle PCR libraries were similar to the naturally occurring fragmentation profiles without amplification, while bin-level GC correction only partially alleviated GC bias in 4 or 12 cycle PCR libraries.

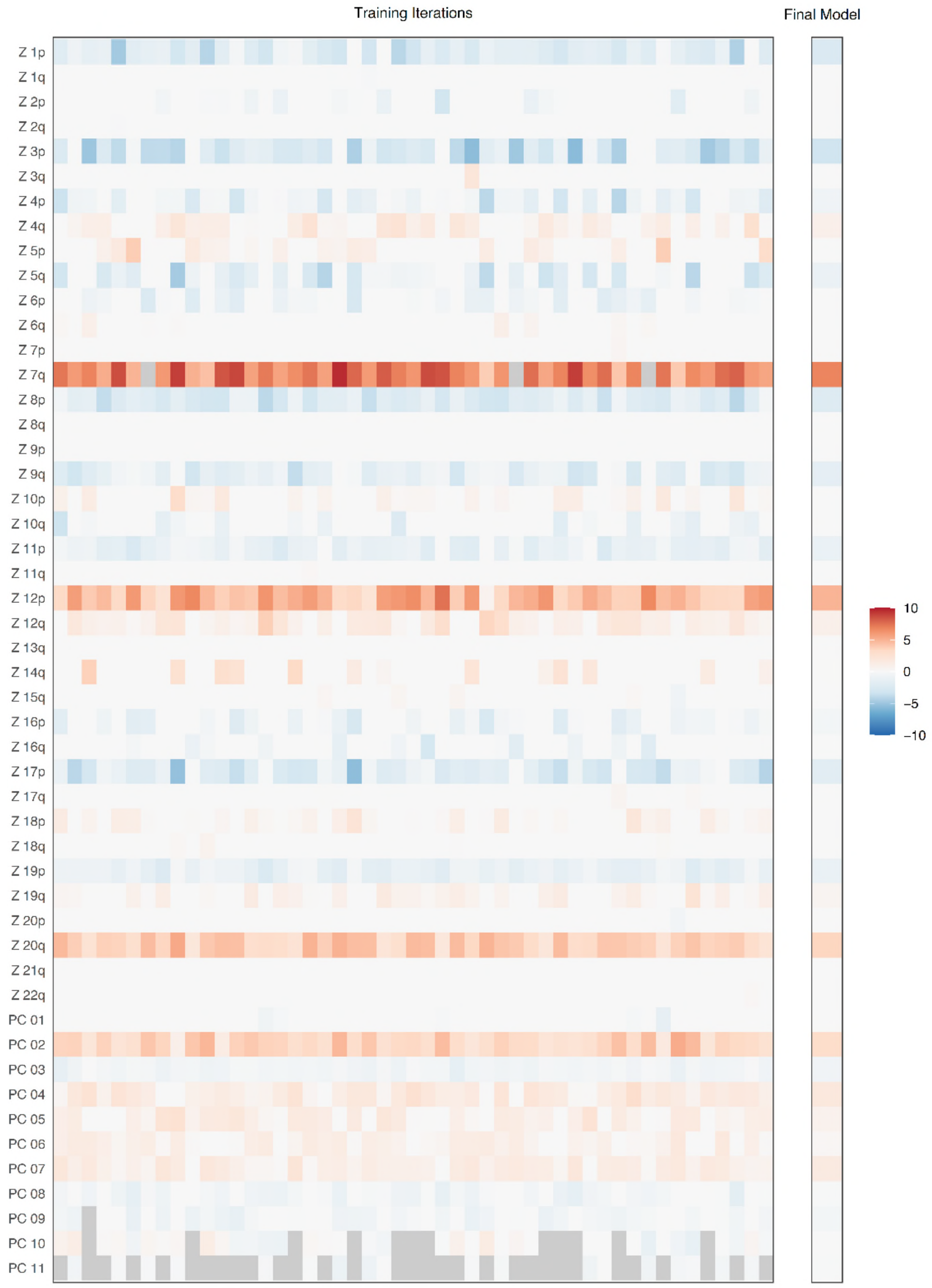

Supplementary Fig. 3. Heatmap representation of the variation of feature contributions to the final DELFI model over 50 training iterations. The heatmap indicates the scaled regression coefficients of each model feature (vertical axis) across the 50 training sets (five-fold x 10 repeats, horizontal axis). The gray boxes indicate models that do not utilize the indicated principal components (PC). The right margin represents the final model used for external validation using the available data from the LUCAS analyses.

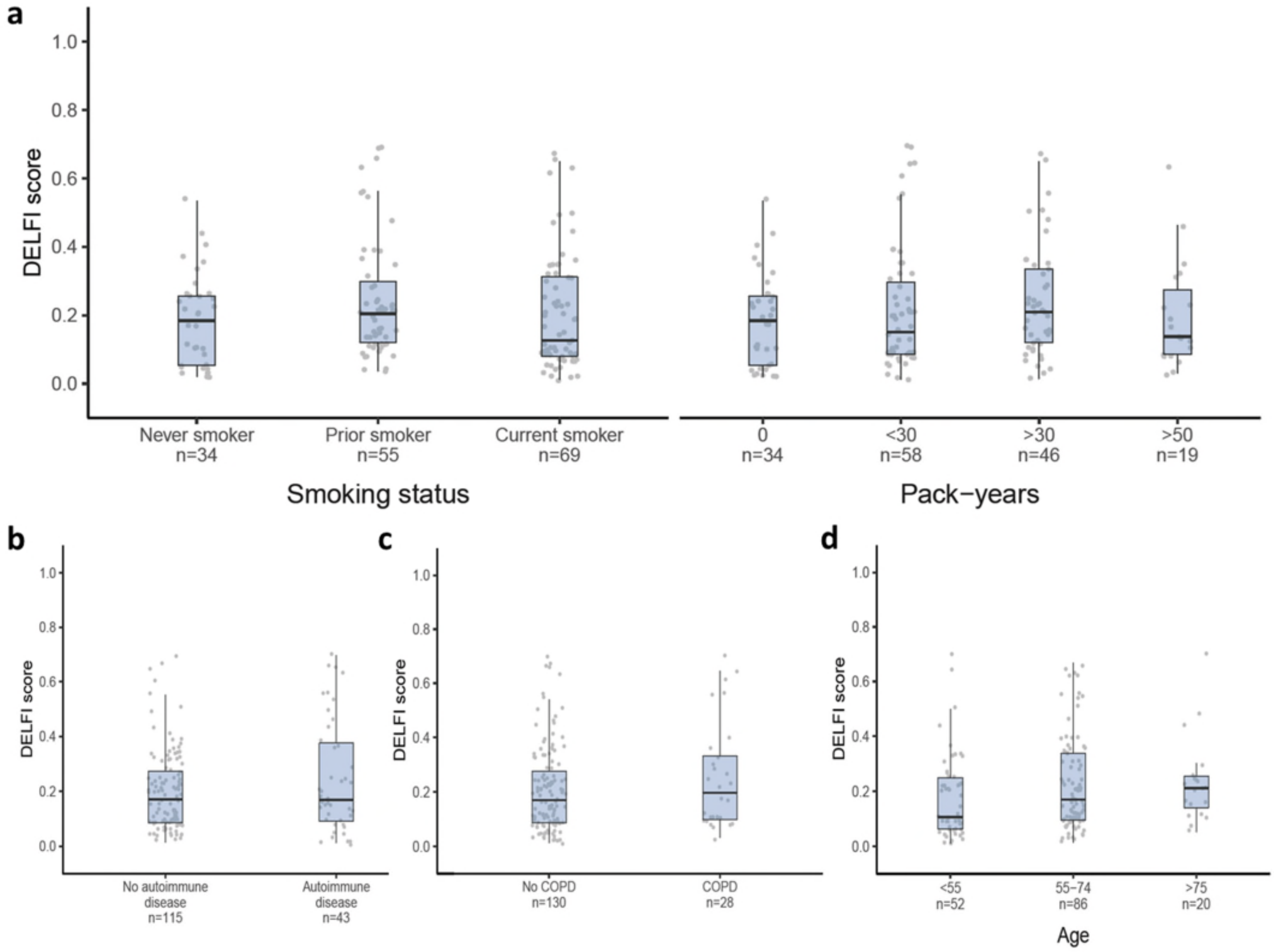

Supplementary Fig. 4. Effect of smoking status, age, and comorbidities on DELFI scores in non-cancer individuals. **a,** The DELFI score in individuals without cancer was similar for those currently smoking versus never smokers or prior smokers (Kruskal-Wallis test, df=154, p=0.47, two-sided), as well as across pack-year groups (Kruskal-Wallis test, df=154, p=0.47, two-sided). **b, c,** Individuals without cancer and a diagnosis of autoimmune diseases or COPD had similar DELFI scores to those individuals without these conditions (independent 2-group Mann-Whitney U test, two-sided, p=0.26, p=0.37 respectively). **d,** DELFI scores were not different among clinically relevant age groups (Kruskal-Wallis, df=2, p=0.18, two-sided). The centre line in the boxplots represents the median, the upper limit of the boxplots represents the third quantile (75th percentile), the lower limit of the boxplots represents the first quantile (25th percentile), the upper whiskers is the maximum value of the data that is within 1.5 times the interquartile range over the 75th percentile, and the lower whisker is the minimum value of the data that is within 1.5 times the interquartile range under the 25th percentile.

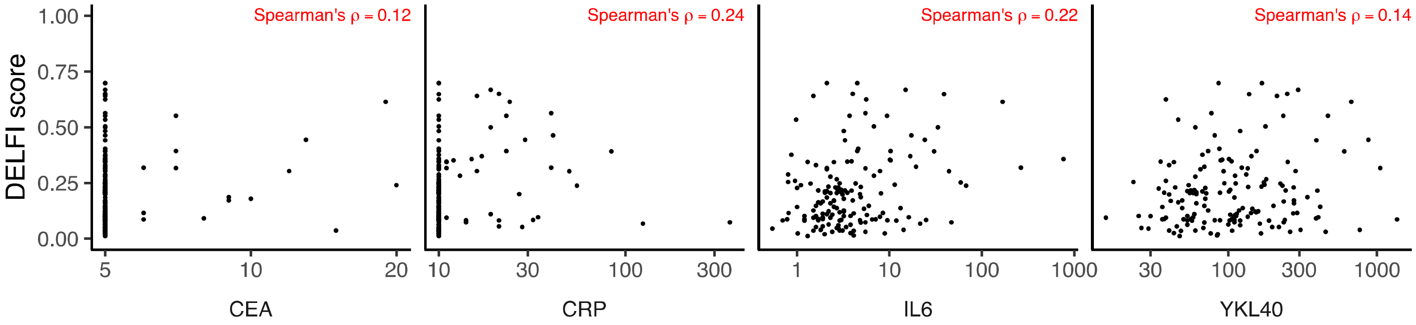

Supplementary Fig. 5. DELFI scores and serum protein markers in non-cancer individuals. Correlation analyses of the DELFI score (vertical axes) with serum protein levels (horizontal axes) in healthy individuals. CRP and YKL-40 levels had weak correlations with DELFI scores (Spearman correlation coefficients: 0.17, p=0.04, two-sided; 0.12, p=0.02, two-sided, respectively). There was no correlation between IL-6 (p=0.08, two-sided) or CEA (p=0.5, two-sided) and DELFI scores.

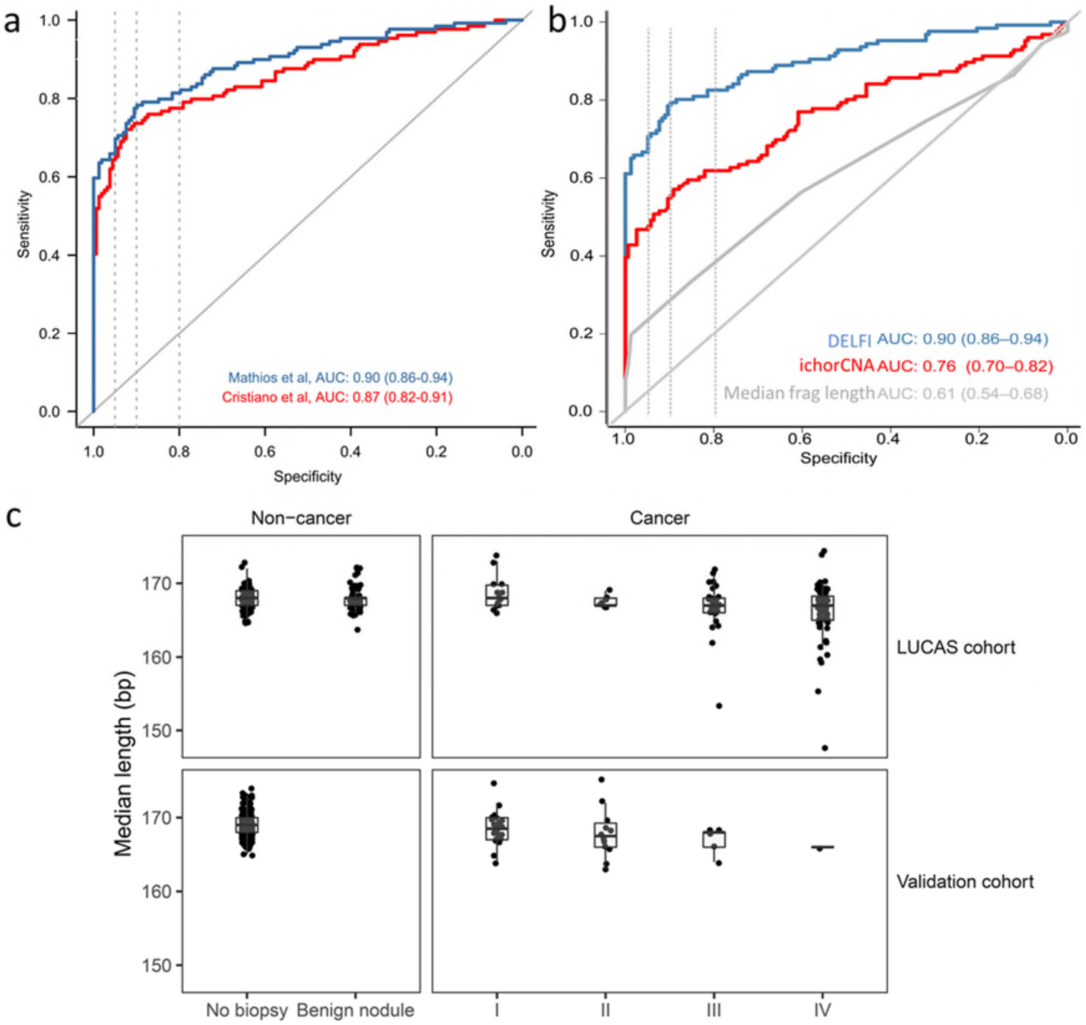

Supplementary Fig. 6. Comparison of performance of DELFI approach with other genomic approaches. a, Implementation of the model features and GC bias correction described in the Cristiano et al. manuscript[23] in the LUCAS cohort (blue) shows a similar performance compared to the model implemented in the current manuscript (red) although the current DELFI model has higher sensitivity at high specificity ranges. **b,** The current DELFI model (blue) outperforms ichor analyses (red) or assessment of median fragment lengths (grey). The dotted vertical lines in the ROC figures represent 95%, 90% and 80% specificities. **c,** Comparison of median fragment sizes by cohort shows similar median fragment lengths for both the LUCAS and the validation cohort for non-cancer individuals as well as patients with cancer separated by stage (n: LUCAS cohort, No biopsy=91 , Validation cohort, No biopsy=385, LUCAS cohort benign nodule=67, LUCAS cohort stage I=15, stage II=7, stage III=35, stage IV=72, Validation cohort stage I=28 stage II=12, stage III=5, stage IV=1). The centre line in the boxplots represents the median, the upper limit of the boxplots represents the third quantile (75th percentile), the lower limit of the boxplots represents the first quantile (25th percentile), the upper whiskers is the maximum value of the data that is within 1.5 times the interquartile range over the 75th percentile, and the lower whisker is the minimum value of the data that is within 1.5 times the interquartile range under the 25th percentile.

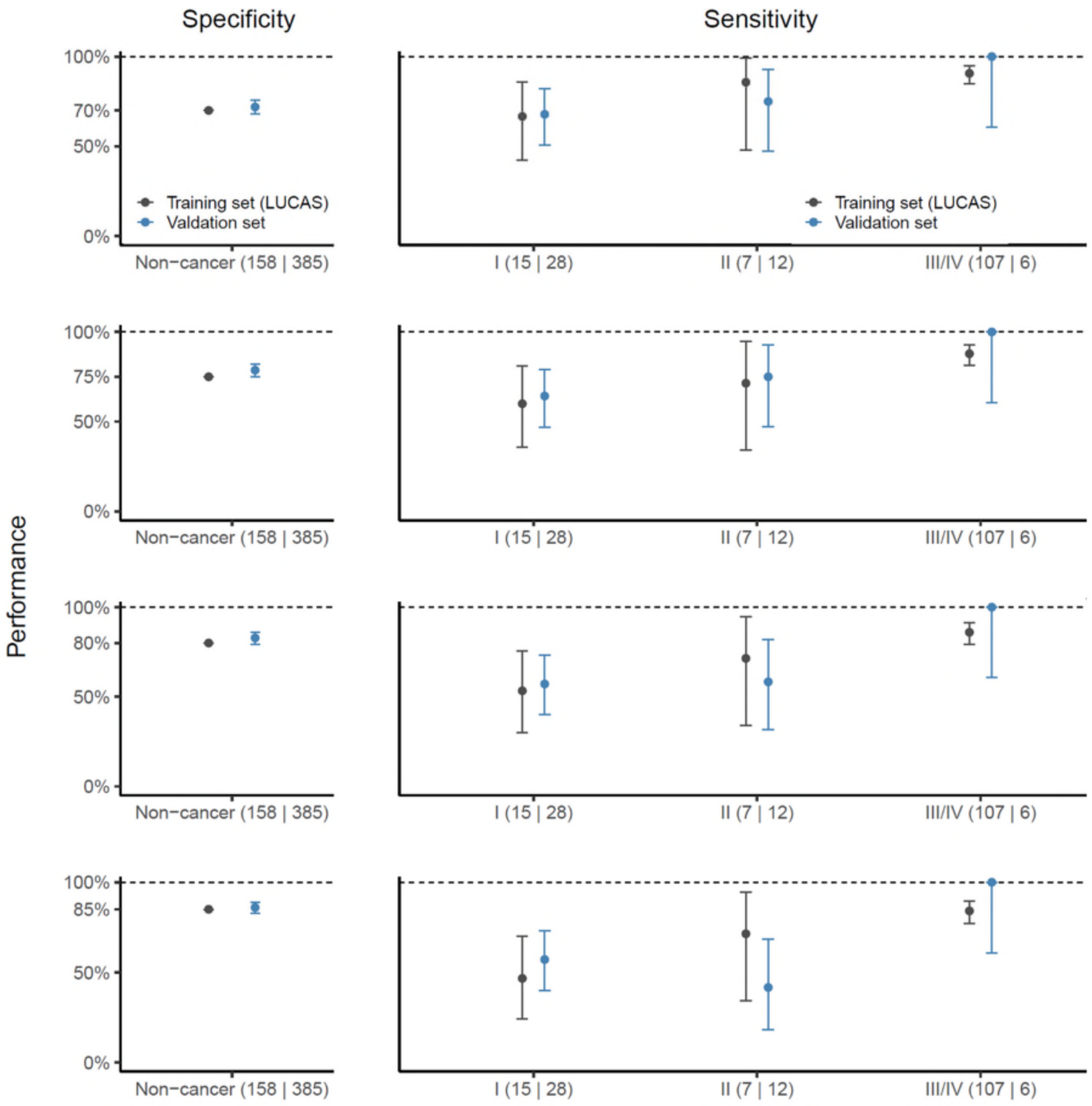

Supplementary Fig. 7. Performance of DELFI in the lung cancer validation cohort. Analysis of the validation cohort comprising 385 non-cancer individuals and 46 lung cancer patients revealed performance metrics similar to the LUCAS cohort across different stages and histological subtypes. The graphs show the specificity and sensitivity of DELFI when a fixed cutoff is used for both the LUCAS and the validation cohort. Each row represents a different DELFI score cutoff (0.252, 0.303, 0.344, or 0.377) that corresponds to a 70%, 75%, 80% or 85% specificity, respectively, as indicated from top to bottom. Median values are indicated with a dot and the intervals presented in the figure reflect a 90% confidence interval.

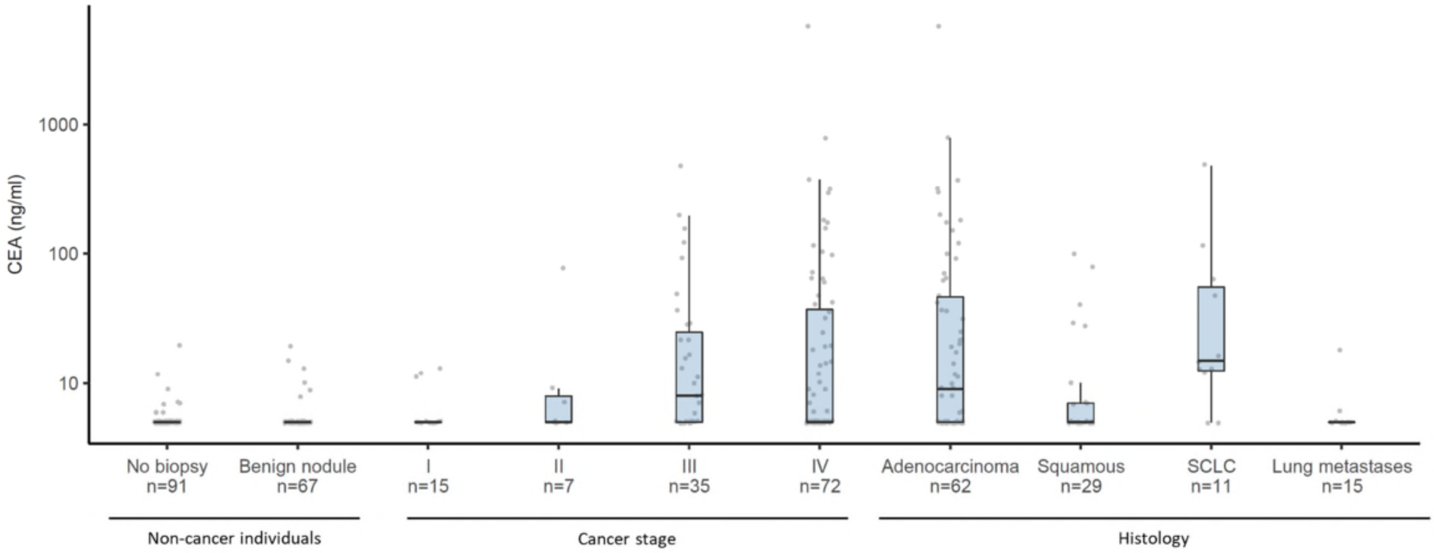

Supplementary Fig. 8. CEA levels across non-cancer individuals and lung cancer patients. Distribution of serum CEA levels (vertical axes) among diagnostic groups, stages, and histological subtypes. The centre line in the boxplots represents the median, the upper limit of the boxplots represents the third quantile (75th percentile), the lower limit of the boxplots represents the first quantile (25th percentile), the upper whiskers is the maximum value of the data that is within 1.5 times the interquartile range over the 75th percentile, and the lower whisker is the minimum value of the data that is within 1.5 times the interquartile range under the 25th percentile.

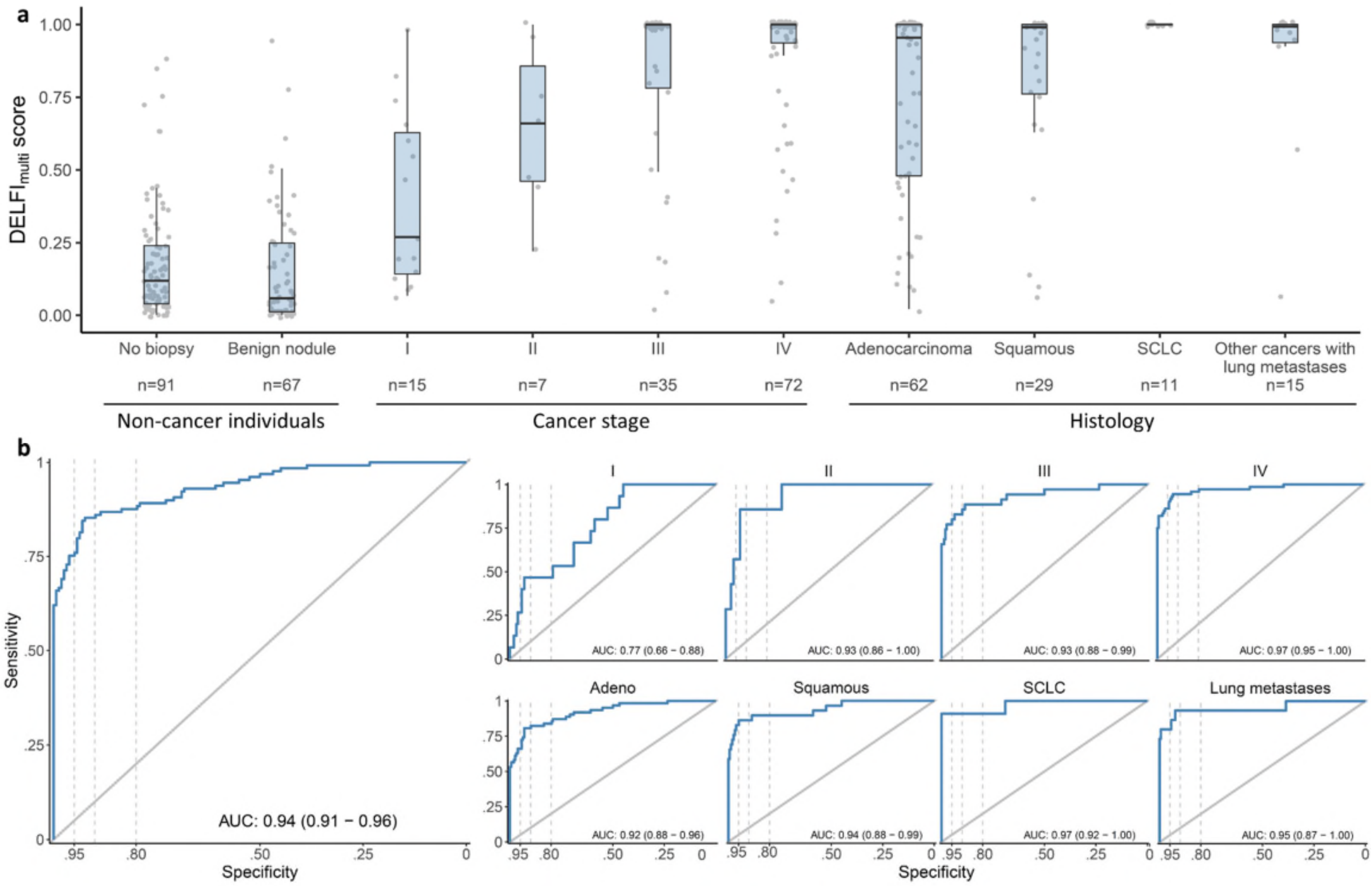

Supplementary Fig. 9. Performance of DELFI$_{multi}$ analyses for lung cancer patients and non-cancer individuals. a, DELFI$_{multi}$ score distribution across stages and histological subtypes. **b,** DELFI ROC curves by stage and histology. The dotted vertical lines in the ROC figures represent 95%, 90% and 80% specificities. Adeno, Adenocarcinoma. The centre line in the boxplots represents the median, the upper limit of the boxplots represents the third quantile (75th percentile), the lower limit of the boxplots represents the first quantile (25th percentile), the upper whiskers is the maximum value of the data that is within 1.5 times the interquartile range over the 75th percentile, and the lower whisker is the minimum value of the data that is within 1.5 times the interquartile range under the 25th percentile.

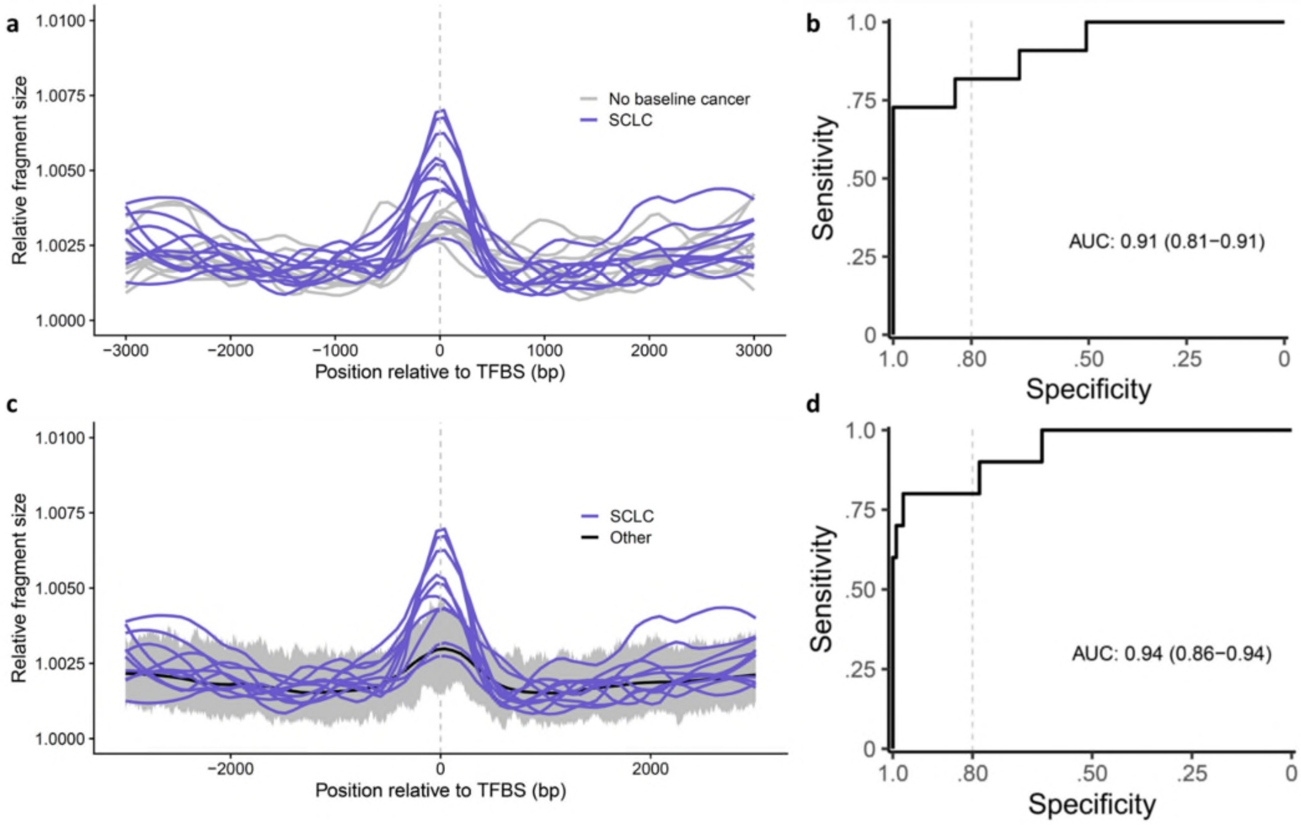

Supplementary Fig. 10. cfDNA fragment sizes at ASCL1 binding sites can distinguish SCLC from non-cancer individuals and NSCLC patients. Genome-wide cfDNA fragmentation analyses at ASCL1 binding sites in LUCAS cohort patients reveals an increase in fragment sizes near transcription factor binding sites of SCLC patients compared to non-cancer individuals (**a**) or individuals with other cancers (**c**). The shaded area in panel c represents the inter-sample variation for individuals without SCLC. This molecular features can distinguish SCLC patients from non-cancer individuals (**b**, AUC=0.91) and SCLC from NSCLC patients (**d**, AUC=0.94), with high accuracy.

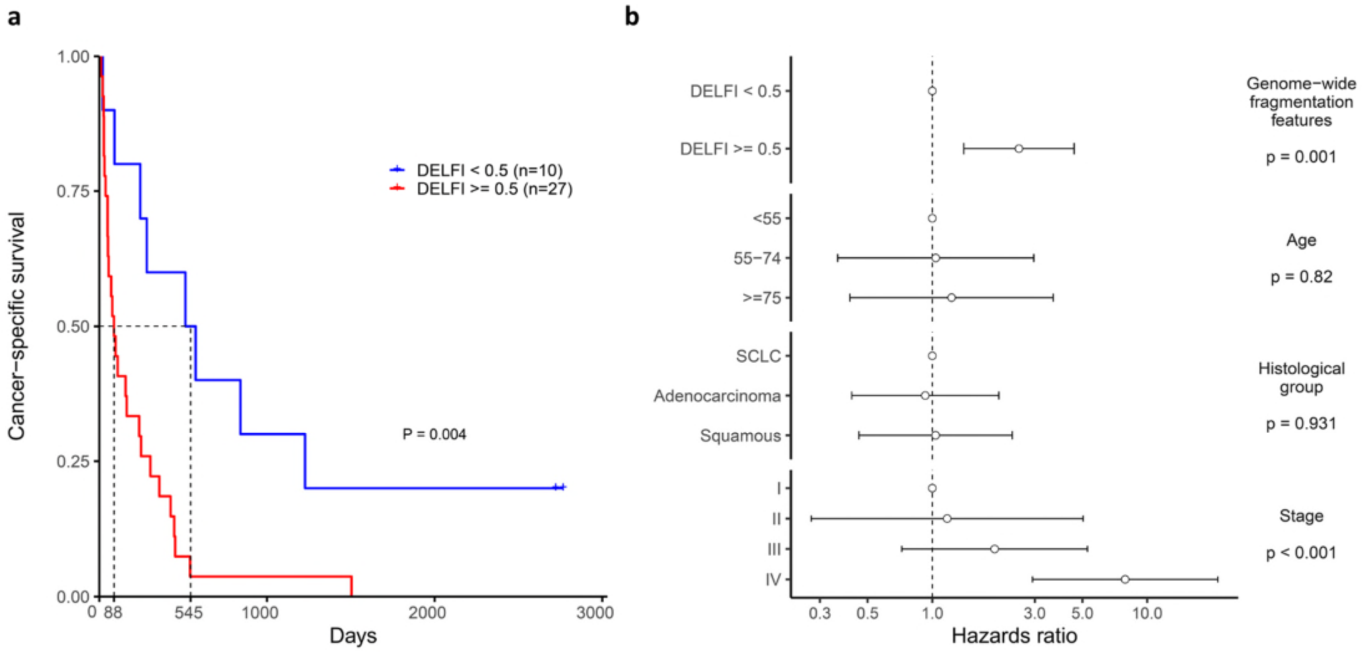

Supplementary Fig. 11. DELFI score and clinical outcome in lung cancer patients. a, Patients with stage IV primary lung cancer with DELFI score <0.5 (blue) revealed a significantly longer cancer-specific survival compared to patients with DELFI scores >0.5 (red) (P=0.004, Log-rank test, 2 sided). **b,** To assess whether the DELFI score was an independent prognostic factor of cancer-specific overall survival we calculated the Cox proportional hazard ratios with high or low DELFI scores, histologic groups, and stage as covariates. Patients with DELFI scores >0.5 had a HR of 2.53 compared to patients with DELFI scores <0.5 (p<0.001, Likelihood ratio test, two -sided) after adjusting for histologic group and stage. The empty circles represent median of hazard ratios and the intervals indicate a 95% confidence interval.

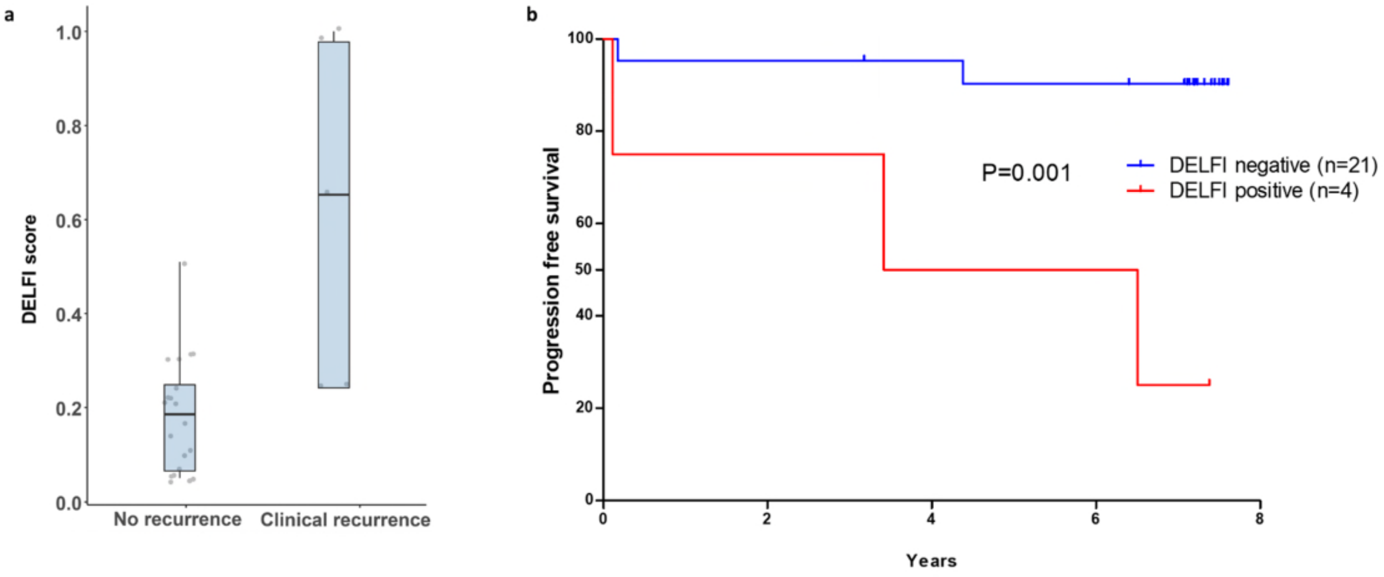

Supplementary Fig. 12 DELFI can identify molecular recurrence prior to clinical recurrence a. Among patients with prior history of cancer, the ones that developed tumor recurrence (n=5) during the follow up period had a significantly higher DELFI score compared to the ones that developed no recurrence (n=20) (independent 2-group Mann-Whitney U test, two-sided, p= 0.004). **b**. Patients with prior history of cancer, no evidence of cancer on baseline assessment and a positive DELFI score (red) (n=4) had a significantly shorter progression-free survival compared to ones with a negative DELFI score (blue, n=21) (p=0.001, Log-rank test, two-sided). The centre line in the boxplots represents the median, the upper limit of the boxplots represents the third quantile (75th percentile), the lower limit of the boxplots represents the first quantile (25th percentile), the upper whiskers is the maximum value of the data that is within 1.5 times the interquartile range over the 75th percentile, and the lower whisker is the minimum value of the data that is within 1.5 times the interquartile range under the 25th percentile.
