# Supplementary material for: Detection and characterization of lung cancer using cell-free DNA fragmentomes
Source: Nat Commun. 2021 Aug 20;12:5060. doi: 10.1038/s41467-021-24994-w (PMC8379179; doi:10.1038/s41467-021-24994-w)
Supplement: Supplementary file 3 — Description of Additional Supplementary Files [file 41467_2021_24994_MOESM3_ESM.pdf]

Description of Additional Supplementary Files

File Name: Supplementary Data 1
Description: Clinical information of LUCAS patients analyzed

File Name: Supplementary Data 2
Description: Summary of whole genome cfDNA anlayses*

File Name: Supplementary Data 3
Description: Patient demographics and clinical information for validation cohort

File Name: Supplementary Data 4
Description: Clinical information of validation cohort patients

File Name: Supplementary Data 5
Description: Serum protein markers in the LUCAS cohort

File Name: Supplementary Data 6
Description: DELFI score and survival analysis in the LUCAS cohort

File Name: Supplementary Data 7
Description: Detection of recurrent cancer in patients with prior history of cancer and no evidence of recurrent disease at baseline

File Name: Supplementary Data 8
Description: Detection of cancer in the LUCAS cohort after baseline assessment

File Name: Supplementary Data 9
Description: Modelling of DELFI performance as a prescreening method to LDCT in a theoritical population

File Name: Supplementary Data 10
Description: Plasma volume and cfDNA amounts used in LUCAS genomic libraries

File Name: Supplementary Data 11
Description: Genomic library controls

File Name: Supplementary Data 12
Description: Chromosomal bin coordinates, GC content, and mappability
