# Supplementary material for: Detection and characterization of lung cancer using cell-free DNA fragmentomes
Source: Nat Commun. 2021 Aug 20;12:5060. doi: 10.1038/s41467-021-24994-w (PMC8379179; doi:10.1038/s41467-021-24994-w)
Supplement: Supplementary file 5 — Reporting Summary [file 41467_2021_24994_MOESM5_ESM.pdf]

nature research

Corresponding author(s): Velculescu V.E., Scharpf R.B.

Last updated by author(s): May 31, 2021

Reporting Summary

Nature Research wishes to improve the reproducibility of the work that we publish. This form provides structure for consistency and transparency in reporting. For further information on Nature Research policies, see our Editorial Policies and the Editorial Policy Checklist.

Statistics

For all statistical analyses, confirm that the following items are present in the figure legend, table legend, main text, or Methods section.

n/a	Confirmed	
☐	☒	The exact sample size (*n*) for each experimental group/condition, given as a discrete number and unit of measurement
☐	☒	A statement on whether measurements were taken from distinct samples or whether the same sample was measured repeatedly
☐	☒	The statistical test(s) used AND whether they are one- or two-sided<br>*Only common tests should be described solely by name; describe more complex techniques in the Methods section.*
☐	☒	A description of all covariates tested
☐	☒	A description of any assumptions or corrections, such as tests of normality and adjustment for multiple comparisons
☐	☒	A full description of the statistical parameters including central tendency (e.g. means) or other basic estimates (e.g. regression coefficient) AND variation (e.g. standard deviation) or associated estimates of uncertainty (e.g. confidence intervals)
☐	☒	For null hypothesis testing, the test statistic (e.g. *F*, *t*, *r*) with confidence intervals, effect sizes, degrees of freedom and *P* value noted<br>*Give P values as exact values whenever suitable.*
☐	☒	For Bayesian analysis, information on the choice of priors and Markov chain Monte Carlo settings
☐	☒	For hierarchical and complex designs, identification of the appropriate level for tests and full reporting of outcomes
☒	☐	Estimates of effect sizes (e.g. Cohen's *d*, Pearson's *r*), indicating how they were calculated

Our web collection on statistics for biologists contains articles on many of the points above.

Software and code

Policy information about availability of computer code

Data collection	All statistical analyses were performed using R version 3.6.1. For processing unaligned reads, we used fastp (v0.20.0), bowtie2 (v 2.3.5.1), samtools (v 1.9), and sambamba (v 0.7.1).
Data analysis	Custom scripts were implemented in R (v 4.0.5) for all subsequent analyses. These scripts are maintained in a GitHub repository (https://github.com/cancer-genomics/reproduce_lucas_wflow) under the GNU GENERAL PUBLIC LICENSE Version 3. All R packages and dependencies from these customized scripts were captured programmatically and made available in the GitHub repository.

For manuscripts utilizing custom algorithms or software that are central to the research but not yet described in published literature, software must be made available to editors and reviewers. We strongly encourage code deposition in a community repository (e.g. GitHub). See the Nature Research guidelines for submitting code & software for further information.

Data

Policy information about availability of data

All manuscripts must include a data availability statement. This statement should provide the following information, where applicable:

- Accession codes, unique identifiers, or web links for publicly available datasets
- A list of figures that have associated raw data
- A description of any restrictions on data availability

Sequence data and clinical variables generated in this study have been deposited at the database of European Genome-Phenome Archive (EGA) under accession code: EGAS00001005340.
The publicly available RNA-seq data used in this study is available in the recount3 database with the project IDs SRP045225, LUAD, LUSC, and BLOOD [https://jhubiostatistics.shinyapps.io/recount3-study-explorer/].
The publicly available ChIP-seq data used in this study is available in the GEO DataSets database under accession code GSM3704421 [https://www.ncbi.nlm.nih.gov/

geo/query/acc.cgi?acc=GSM3704421]. Segmented copy number data, determined by analysis of the Affymetrix genome-wide human SNP array 6.0, were retrieved from Broad Institute TCGA Genome Data Analysis Center, (2016-01-28 release date, using RTCGA package, version 1.16.0). The remaining data are available within the Article, Supplementary Information or Source Data file.

Field-specific reporting

Please select the one below that is the best fit for your research. If you are not sure, read the appropriate sections before making your selection.

☒ Life sciences ☐ Behavioural & social sciences ☐ Ecological, evolutionary & environmental sciences

For a reference copy of the document with all sections, see nature.com/documents/nr-reporting-summary-flat.pdf

Life sciences study design

All studies must disclose on these points even when the disclosure is negative.

Sample size	The prevalence of undiagnosed cancer cases in this prospectively collected cohort was expected to be high (approximately 30 cancers per 100 non-cancer individuals). Assuming a noninvasive test for cancer with a specificity of 0.85 in a study of nearly 400 participants, our study would provide an estimate of sensitivity with a margin of error of 0.05 or smaller.
Data exclusions	3 subjects were excluded due to failure in the sequencing step and no data were able to be retrieved from the sequencer, therefore no data were available for analysis. The above is a pre-established requirement for inclusion of samples in the the cohorts analyzed.
Replication	We replicated our findings in a cohort of 431 independently collected and processed samples.
Randomization	Cancer and non-cancer individuals were prospectively collected in this cohort. Every library preparation batch and cfDNA extraction batch included cancer and non-cancer individuals. Due to the prospective study design and the random allocation of cancer and non-cancer samples to each batch, we expect that both known and unknown confounders would be similar across the collection of batches.
Blinding	The investigators were not blinded to diagnosis for the LUCAS cohort since this cohort was used for training of the machine learning model. While the investigators that prepared the libraries for the validation cohort had to know the diagnosis of each individual used to randomize the samples across library preparation, the person that performed the machine learning model development was blinded to diagnosis of the validation cohort.

Reporting for specific materials, systems and methods

We require information from authors about some types of materials, experimental systems and methods used in many studies. Here, indicate whether each material, system or method listed is relevant to your study. If you are not sure if a list item applies to your research, read the appropriate section before selecting a response.

Materials & experimental systems

n/a	Involved in the study	
☒	☐	Antibodies
☒	☐	Eukaryotic cell lines
☒	☐	Palaeontology and archaeology
☒	☐	Animals and other organisms
☐	☒	Human research participants
☒	☐	Clinical data
☒	☐	Dual use research of concern

Methods

n/a	Involved in the study	
☒	☐	ChIP-seq
☒	☐	Flow cytometry
☒	☐	MRI-based neuroimaging

Human research participants

Policy information about studies involving human research participants

Population characteristics	The LUCAS cohort consisted of patients of age 19-96. There were 186 male patients and 179 female patients. The validation cohort consisted of patients of age 38-76. There were 210 male patients and 221 female patients. There were 236 individuals with no-baseline cancer and 129 individuals with baseline lung cancer in the LUCAS cohort. Out of the 236 individuals with no-baseline cancer, 183 had no prior history of cancer and the rest 53 had a prior history of cancer that was in remission at the time of baseline assessment. For the validation cohort none of the individuals included had a history of prior cancer. Treatment for the patients with lung cancer in the LUCAS cohort was performed in accordance with the most updated ESMO guidelines at the time of treatment.
Recruitment	The LUCAS cohort represents a prospectively collected group of 368 consecutive patients age >18 that presented in the Department of Respiratory Medicine, Infiltrate Unite, Bispebjerg Hospital, Copenhagen with a positive imaging finding on a chest X-ray or a chest CT. Patients with known cancer and active disease or who were undergoing treatment at the time of enrollment were excluded. The collection lasted from September 2012 to March of 2013. The validation cohort consisted of 385 non-cancer individuals from two screening cohorts for colorectal cancer in Denmark (Endoscopy III observational clinical trial) and the Netherlands (COCOS observational clinical trial). The inclusion criteria for both studies were patients age 50-75 eligible for colorectal cancer screening. The patients analyzed in the manuscript had to have either a negative FIT test or a negative colonoscopy result. 46 patients at risk for lung cancer at the time of blood collection that were identified upon further diagnostic workup to have a new diagnosis of pathologically confirmed predominantly early stage lung cancer from an independent prospective collection through BioIVT (Westbury, NY) were included in the study. The above collections while well curated have potential biases. The LUCAS cohort consists of patients with predominantly symptomatic disease, although most symptoms are common symptoms that the majority of patients in a screening program would have (such as cough, sputum production, dyspnea). It is therefore possible that there is a selection bias introduced. For the COCOS and Endoscopy III collections while we have assured that the patients do not have colorectal cancer it is possible they could have other undiagnosed cancer types at the time of enrollment. Additionally, patients enrolling in a colorectal cancer screening are not necessarily matching the population screened for lung cancer and therefore this could potentially introduce a bias in the analysis. The lack of full clinical and follow-up data in both the COCOS and Endoscopy III cohorts as well as the BioIVT collections does not allow us to fully assess contribution of clinical characteristics in performance of the prediction model presented here.
Ethics oversight	The protocol for the Endoscopy III Project has been approved by the Regional Ethics Committee (H-4-2013-050) and the Danish Data Protection Agency (2007-58-0015/HVH-2013-022). The LUCAS study was performed according to the declaration of Helsinki and approved by the Danish Regional Ethics Committee (H-2-2011-147) and the Danish Data Protection Agency (j.nr. 2012-58-0004; HEH 750.24.56 and HGH-2018-017; I-Suite nr. 6215). For the COCOS trial ethics approval was obtained from the Dutch Health Council (2009/03WBO, The Hague, Netherlands). The BioIVT samples were purchased from the company de-identified and are not considered human subjects research as they are deidentified.

Note that full information on the approval of the study protocol must also be provided in the manuscript.
