# Supplementary material for: Detection and characterization of lung cancer using cell-free DNA fragmentomes
Source: Nat Commun. 2021 Aug 20;12:5060. doi: 10.1038/s41467-021-24994-w (PMC8379179; doi:10.1038/s41467-021-24994-w)
Supplement: Supplementary file 2 — Peer Review File [file 41467_2021_24994_MOESM2_ESM.pdf]

Editorial Note: This manuscript has been previously reviewed at another journal that is not operating a transparent peer review scheme. This document only contains reviewer comments and rebuttal letters for versions considered at Nature Communications.

Reviewers' Comments:

Reviewer #1:
Remarks to the Author:
I thank the authors for their response to the comments. I find the manuscript improved and providing greater clarity with regards to strengths and weaknesses. This paper is an important contribution to the field.

Reviewer #2:
Remarks to the Author:
The authors have now satisfactorily addressed all concerns.

Detailed Reponses to Referee Comments

Manuscript ID: NCOMMS-21-16407-T

Detection and characterization of lung cancer using cell-free DNA fragmentomes

REVIEWERS' COMMENTS

Reviewer #1 (Remarks to the Author):

I thank the authors for their response to the comments. I find the manuscript improved and providing greater clarity with regards to strengths and weaknesses. This paper is an important contribution to the field.

Response: We thank the reviewer for the positive feedback.

Reviewer #2 (Remarks to the Author):

The authors have now satisfactorily addressed all concerns.

Response: We appreciate the reviewer's satisfaction with the revisions.
